# Pharmacological Elevation of Catecholamine Levels Improves Perceptual Decisions, But Not Metacognitive Insight

**DOI:** 10.1523/ENEURO.0019-24.2024

**Published:** 2024-07-26

**Authors:** Stijn A. Nuiten, Jan Willem de Gee, Jasper B. Zantvoord, Johannes J. Fahrenfort, Simon van Gaal

**Affiliations:** ^1^Department of Psychology, University of Amsterdam, Amsterdam, Netherlands; ^2^Amsterdam Brain & Cognition, University of Amsterdam, Amsterdam, Netherlands; ^3^Department of Psychiatry (UPK), University of Basel, Basel, Switzerland; ^4^Cognitive and Systems Neuroscience, Swammerdam Institute for Life Sciences, University of Amsterdam, Amsterdam, Netherlands; ^5^Department of Psychiatry, Amsterdam UMC location AMC, Amsterdam, Netherlands; ^6^Amsterdam Neuroscience, Amsterdam, Netherlands; ^7^Institute for Brain and Behavior Amsterdam, Vrije Universiteit Amsterdam, Amsterdam, Netherlands; ^8^Department of Experimental and Applied Psychology - Cognitive Psychology, Vrije Universiteit Amsterdam, Amsterdam, Netherlands

**Keywords:** acetylcholine, arousal, catecholamines, EEG, metacognition, perception

## Abstract

Perceptual decisions are often accompanied by a feeling of decision confidence. Where the parietal cortex is known for its crucial role in shaping such perceptual decisions, metacognitive evaluations are thought to additionally rely on the (pre)frontal cortex. Because of this supposed neural differentiation between these processes, perceptual and metacognitive decisions may be divergently affected by changes in internal (e.g., attention, arousal) and external (e.g., task and environmental demands) factors. Although intriguing, causal evidence for this hypothesis remains scarce. Here, we investigated the causal effect of two neuromodulatory systems on behavioral and neural measures of perceptual and metacognitive decision-making. Specifically, we pharmacologically elevated levels of catecholamines (with atomoxetine) and acetylcholine (with donepezil) in healthy adult human participants performing a visual discrimination task in which we gauged decision confidence, while electroencephalography was measured. Where cholinergic effects were not robust, catecholaminergic enhancement improved perceptual sensitivity, while at the same time leaving metacognitive sensitivity unaffected. Neurally, catecholaminergic elevation did not affect sensory representations of task-relevant visual stimuli but instead enhanced well-known decision signals measured over the centroparietal cortex, reflecting the accumulation of sensory evidence over time. Crucially, catecholaminergic enhancement concurrently impoverished neural markers measured over the frontal cortex linked to the formation of metacognitive evaluations. Enhanced catecholaminergic neuromodulation thus improves perceptual but not metacognitive decision-making.

## Significance Statement

Perceptual decisions about sensory input and the metacognitive evaluation of the accuracy of such decisions may be inversely affected by neuromodulatory systems regulating organisms’ arousal levels. We tested this hypothesis by pharmacologically manipulating two neuromodulator systems (catecholaminergic, cholinergic) in humans, while measuring EEG. Elevated levels of catecholamines, but not acetylcholine, increased the accuracy of perceptual decisions, but not the accuracy of metacognitive evaluations thereof. Further, catecholamines enhanced neural markers over the parietal cortex associated with the accumulation of evidence used for perceptual decision-making, while perturbing markers of metacognitive decision-making over the frontal cortex. These findings align with current theories of perceptual decision-making, metacognition, and cortical functioning and improve our understanding of the important role of neuromodulation in shaping human behavior and cognition.

## Introduction

Humans process, discriminate, and categorize sensory information from their surroundings to meaningfully guide behavior, a process referred to as perceptual decision-making (Heekeren et al., 2008; Hanks and Summerfield, 2017; O’Connell and Kelly, 2021). Such perceptual decisions are often accompanied by a feeling of decision confidence, which aids learning and adaptive behavior (Yeung and Summerfield, 2012; Zylberberg et al., 2012; Fleming et al., 2012b; Meyniel et al., 2015). In recent years, several classes of models were developed to explain how metacognitive evaluations of task performance arise during perceptual decision-making (Fleming et al., 2012a; Mamassian, 2016; Rahnev et al., 2022; Webb et al., 2023). For example, single-channel models propose that perceptual and metacognitive decisions are based on the same underlying information (Del Cul et al., 2009; Hangya et al., 2016; Sanders et al., 2016). In contrast, under parallel (or dual-channel) and hierarchical models, perceptual and metacognitive decisions are corrupted by distinct sources of (inference) noise and hence based on different information, further suggesting that these processes may be dissociable (Meyniel et al., 2015; Fleming and Daw, 2017; J. W. Bang et al., 2019). Indeed, there is convergent empirical work demonstrating that under certain experimental conditions, perceptual and metacognitive decision-making behavior do not always scale proportionally and can be independently manipulated (Wilimzig et al., 2008; Sherman et al., 2015; Maniscalco et al., 2016; Spence et al., 2018; Shekhar and Rahnev, 2021a,b).

Neural recordings further support the idea that these decision types are separable, as perceptual decisions and metacognitive evaluations seem to rely on different cortical networks (Di Luzio et al., 2022). More specifically, perceptual decision-making is associated with activity in (centro)parietal regions that is deemed responsible for the integration of sensory information into a decision variable, reflecting the evidence for the upcoming decision (Gold and Shadlen, 2007; Twomey et al., 2015). This sensory evidence is accumulated over time until it crosses a certain internal threshold, upon which a decision is made. In humans, sensory evidence accumulation is thought to be reflected in an event-related potential (ERP) measured over centroparietal regions, referred to as the centroparietal positivity (CPP; O’Connell et al., 2012). Neuroimaging and transcranial magnetic stimulation (TMS) studies have additionally implicated the (pre)frontal cortex in the formation of metacognitive decisions (Rounis et al., 2010; Fleming et al., 2012b; D. Bang and Fleming, 2018; Di Luzio et al., 2022). For instance, metacognitive performance is selectively impaired (compared with perceptual performance) when interfering with dorsolateral prefrontal activity through TMS (Rounis et al., 2010). Although there is still a lot to learn about the exact computations in frontal networks underlying metacognitive evaluations, recent work has identified the frontal potential (FP) ERP component as a marker of metacognitive evaluations, as it tracks upcoming decision confidence (Lim et al., 2020), on correct trials (Feuerriegel et al., 2022).

Recent empirical work also suggests that the arousal state of an organism, driven by neuromodulator systems (Aston-Jones and Cohen, 2005; McCormick et al., 2020), may differentially affect perceptual and metacognitive decision-making. For example, pharmacological elevation of baseline levels of catecholamines (i.e., noradrenaline and dopamine) improves perceptual sensitivity (*d*’; Gelbard-Sagiv et al., 2018; Nuiten et al., 2023), whereas antagonizing catecholaminergic receptors selectively improves metacognitive sensitivity (meta-*d*’; Hauser et al., 2017). This suggests that perceptual and metacognitive processing are oppositely affected by fluctuations in (catecholaminergic) neuromodulator levels. Catecholamines have further been shown to modulate the CPP component, reflecting evidence accumulation processes (Loughnane et al., 2019; Nuiten et al., 2023). However, if and how neuromodulatory drive modulates activity related to metacognition is not known [although neuromodulatory systems are known to strongly innervate the (pre)frontal cortex; Cools and Arnsten, 2022]. Moreover, it is currently unclear whether neuromodulation selectively modulates centroparietal evidence accumulation processes or whether “downstream” encoding of sensory information is also affected (Warren et al., 2016). Finally, it has not been established whether solely the catecholaminergic system is related to metacognitive decision-making or if other neuromodulatory systems are also involved.

Here we addressed these gaps in the literature and investigated if and how increasing catecholaminergic (through atomoxetine, ATX) and cholinergic (through donepezil, DNP) neuromodulator levels affected behavioral and neural markers of perception and metacognitive insight, compared with a placebo condition (PLC). Specifically, we asked (1) whether pharmacological elevation of catecholaminergic and cholinergic levels differentially affects perceptual versus metacognitive sensitivity, (2) whether neuromodulatory effects on perceptual decision-making behavior are related to altered neural representations of sensory input, sensory evidence accumulation (CPP component) or both, and (3) whether neural markers of metacognitive decision-making (FP component) are affected by elevated neuromodulator levels.

## Materials and Methods

### Participants

For this experiment, 30 healthy male human participants were recruited from the online research environment from the University of Amsterdam. All participants were aged between 18 and 30 years. Given the pharmacological nature of this experiment, participants were only included after passing a physical and mental screening (see below, Screening procedure). This study was approved by the Medical Ethical Committee of the Amsterdam Medical Centre (AMC) and the local ethics committee of the University of Amsterdam. Written informed consent was obtained from all participants after explanation of the experimental protocol. Two participants decided to withdraw from the experiment after having performed the first experimental session. The data from these participants are not included in this work. One other participant performed near ceiling on the perceptual task and had a perceptual sensitivity (*d*’, averaged across experimental sessions) that exceeded >2.5 standard deviations of the population mean of *d*’ scores. Furthermore, this participants’ metacognitive sensitivity (meta-*d*’; see Materials and Methods) was <0 on all experimental sessions, suggesting that this participant consistently pressed the incorrect buttons to indicate decision confidence. Therefore, we excluded this participant from all analyses resulting in a final dataset containing *N* = 27 participants. All participants received a monetary compensation for participation in this study.

### Screening procedure

Following registration, candidate participants were contacted via email in which they were informed on the inclusion criteria, exact procedures, and possible risks. Moreover, we provided the credentials of an independent physician that was available for questions and further explanation. The participants were contacted after a minimal deliberation period of 7 d, to verify whether they were still interested in participation. If so, participants were invited for a preintake via telephone. During this preintake, it was ensured that the candidate participant indeed met all inclusion and none of the exclusion criteria. If so, participants were invited for an intake session at the research facility of the University of Amsterdam. During this physical intake, the experimental protocol, including the following physical and mental assessment, was explained in detail after which candidate participants provided written consent. The intake consisted of a set of physiological measures (BMI, heart rate, and blood pressure), an electrocardiogram (ECG), a medical history, and a psychiatric questionnaire to assess mental health status and history. The data from the intake were assessed by a physician, who subsequently decided whether a candidate participant was eligible for participation. Participants with a current or history of neurological disease or mental disorders (including substance use disorders) and participants with a first-degree relative with schizophrenia or major depression were excluded. The use of psychotropic medication or recreational drugs were not permitted 72 h (24 h for alcohol) prior to each test session.

### Drug administration

**Figure 1.**
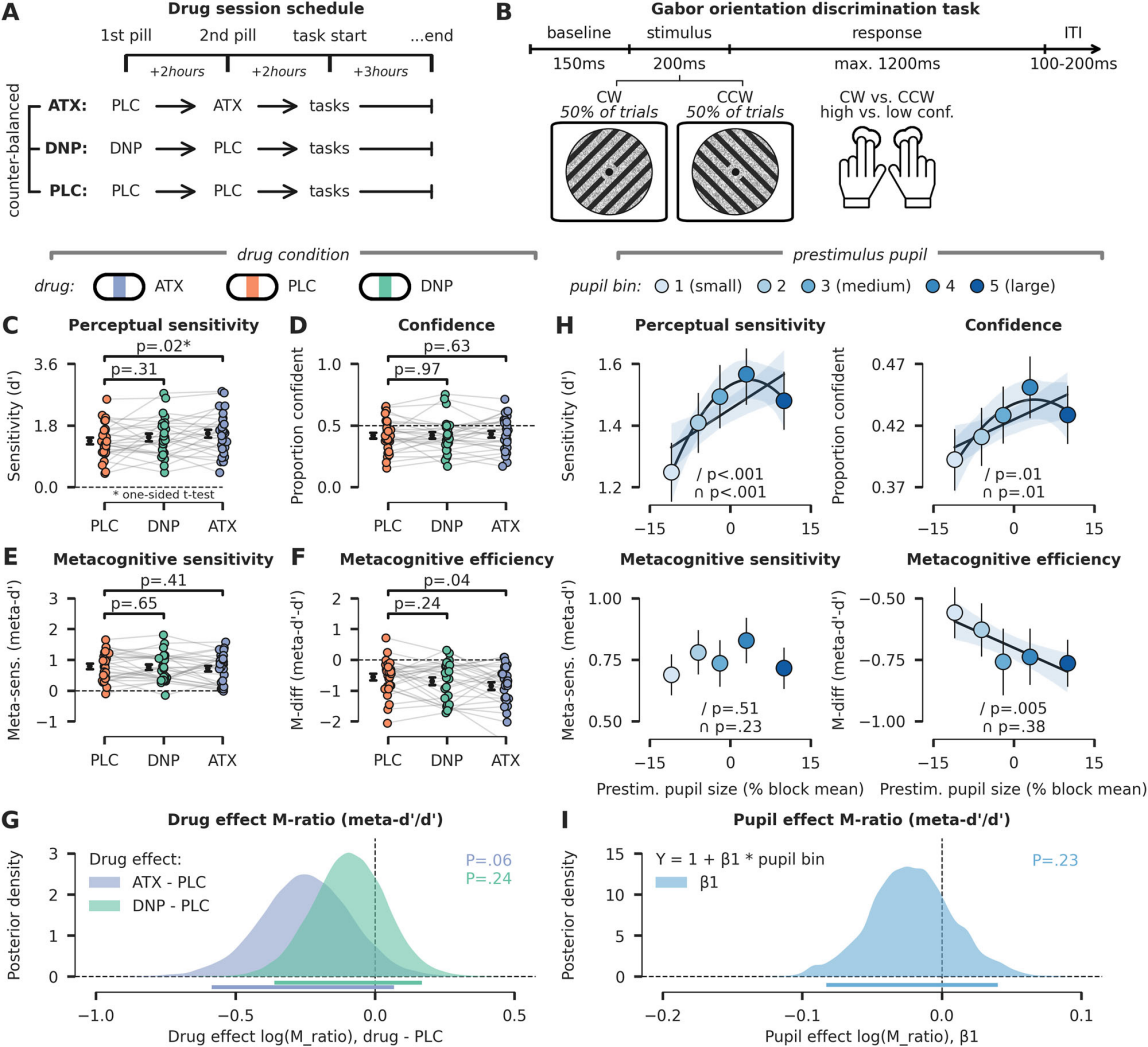
Experimental setup and behavioral effects of pharmacological and pupil-linked elevations of neuromodulatory activity. ***A***, Schematic overview of experimental sessions. Participants came to the lab on four occasions: one intake session and three experimental sessions. On the experimental sessions, participants received either placebo (PLC, data in orange), donepezil (DNP, 5 mg, data in green), or atomoxetine (ATX, 40 mg, data in blue). Drug order was counterbalanced across participants. To account for differences in pharmacokinetics (ATX takes ∼2 h to reach peak plasma level, DNP ∼4 h), while keeping participants blind for the drug conditions, we administered two pills at two fixed times on every session. The first pill (administered at the beginning of the session) contained either PLC (for PLC and ATX session) or DNP (DNP session), and the second pill (administered 2 h later) was either a PLC (for PLC and DNP session) or ATX (ATX session). Behavioral testing started 4 h after administration of the first pill. ***B***, Schematic representation of the behavioral task. Participants reported the orientation of a centrally presented Gabor patch as clockwise (CW) versus counterclockwise (CCW). At the same time, participants provided a binary report on how confident they were in their perceptual judgment (high confidence or low confidence). ***C***, Perceptual sensitivity (*d*’) for all drug conditions and confidence levels. ATX, but not DNP, significantly increased *d*’ compared with PLC. ***D***, The proportion of high confident answers was not modulated by drug condition. ***E***, Metacognitive sensitivity (meta-*d*’) was not modulated by drug condition. ***F***, Metacognitive efficiency (*M*-diff, meta-*d*’ − *d*’) was lower under ATX as compared with PLC. ***G***, Hierarchical Bayesian estimation of *M*-ratio (meta-*d*’ / *d*’). Density plots show posterior distributions for drug effects (drug—PLC) on log-transformed *M*-ratio scores. Log-transformed values below zero are indicative of a reduction in *M*-ratio. Horizontal bars indicate the 95% credibility intervals for the ATX effect (blue) and DNP effect (green). The reported *p* value (note: capital letter) is the probability that the true drug effect is greater than zero. ***H***, Same as ***C–F***, but for prestimulus pupil size bins, ranging from relatively small (light blue) to relatively large (dark blue). Linear (indicated with /) and quadratic (indicated with ∩) regression fits are plotted when significant. Note that the *x*-axis depicts the average pupil diameter of each bin (in % of block mean), but that the regression analyses were performed on bin number (1–5). ***I***, Same as ***G***, but here the density plot indicates the posterior distribution for beta weights describing a linear relation between prestimulus pupil size bin and *M*-ratio. Error bars indicate standard error of the mean (SEM).

The pharmaceuticals used in this study were chosen on the basis of their pharmacokinetic and pharmacodynamic properties as well as the relatively limited side effects and prior use of these pharmaceuticals in other studies in the cognitive sciences (Boucart et al., 2015; Warren et al., 2016; Gratton et al., 2017; Pfeffer et al., 2018, 2021). ATX is a relatively selective noradrenaline reuptake inhibitor, which inhibits the presynaptic noradrenaline reuptake transporter, thereby resulting in increased noradrenaline and dopamine levels in the synaptic cleft (Simpson and Plosker, 2004). The half-life of ATX varies between 4.5 and 19 h, and peak plasma levels are reached ∼2 h after administration. DNP is a cholinesterase inhibitor, which impedes the breaking down of acetylcholine by cholinesterase, thereby resulting in increased acetylcholine levels in the synaptic cleft. The elimination half-life of donepezil is 70 h, and peak plasma levels are reached after ∼4 h (Rogers and Friedhoff, 1998). Given the different durations after which DNP (∼4 h) and ATX (∼2 h) reach peak plasma level in the blood, these pharmaceuticals were administered at different times prior to the onset of the behavioral tasks (Fig. 1*A*). To ensure the double-blind design, participants were therefore required to orally ingest a pill 4 h prior to the onset of the first behavioral task, which could contain either DNP or a placebo, and a second pill 2 h prior to onset of the first behavioral task, which could contain either ATX or a placebo. Thus, participants received one placebo and a working pharmaceutical or two placebos on every experimental session.

### Design and procedures

#### Experimental setting

Participants performed several tasks during a single experimental day. The first task was an auditory discrimination/detection task that was executed directly after the administration of the first pill. The data of that experiment fall outside the scope of this paper. An EEG apparatus was connected 3.5 h after ingestion of the first pill, ensuring that participants could start the main experiment precisely 4 h after ingestion of the first pill, at the time when blood concentration levels for both ATX and DNP were projected to be peaking. During the main experiment, participants performed five different computerized visual perception tasks, out of which we will only discuss the current behavioral paradigm. The order of these behavioral tasks was counterbalanced between participants but maintained over sessions. Participants were seated in a darkened, sound-isolated room, 80 cm from a 69 × 39 cm screen using a chinrest (frequency, 60 Hz; resolution, 1,920 × 1,080). The main task and staircase procedure were programmed in Python 2.7 using PsychoPy (Peirce, 2007) and custom-made scripts.

#### Orientation discrimination paradigm

Participants performed a visual orientation discrimination task for which they had to report the orientation of Gabor patches as being clockwise (45°) or counterclockwise (−45°) and simultaneously indicate the confidence in their decision as being high or low. Counterclockwise answers were given with the left hand, clockwise answers with the right hand, and for both hands high confidence was indicated with the middle finger and low confidence with the index finger. So, e.g., high confidence clockwise responses were given with the right middle finger. By having participants simultaneously report Gabor orientation and decision confidence, we ensured that the perceptual and metacognitive decision-making processes were equally lengthy. This was important because a temporal decay of accumulated sensory evidence may affect postdecisional confidence formation (Navajas et al., 2016). Moreover, it has been shown that using concurrent perceptual and metacognitive responses significantly improves the reliability (i.e., test–retest) of metacognitive efficiency measures, when compared with separated perceptual and metacognitive responses (Guggenmos, 2021). Thus, we constrained potential differences between perceptual and metacognitive decision-making processes by using concurrent responses, allowing us to better compare drug effects on perceptual and metacognitive decision-making.

We did not specifically instruct participants to respond as fast or as accurate as possible but did ask them to try to equally (50/50) distribute their confidence reports to prevent participants responding low confidence too often during this challenging task. The Gabor patches were centrally presented for 200 ms (radius, 8.5°; spatial frequency, 1.365 cycles/degree) on top of a circular patch containing dynamic noise (radius, 9.5°). The difficulty of the task was regulated by increasing or decreasing the opacity of the Gabor patch. The opacity was determined for every participant during the intake session and was then fixed over all experimental sessions (for details, see below, Staircasing procedure). The response window started concurrently with stimulus presentation and lasted 1,400 ms. If participants did not respond during this window, they would receive visual feedback informing them of their slow response (in Dutch “te laat,” which translates into “too late”). A variable intertrial interval (ITI) of 250–350 ms started directly after a response or at the end of the response window. CW and CCW stimuli occurrences were balanced 50%.

To ensure participants did not lose fixation or blink during the critical phases of each behavioral trial (stimulus presentation and response window), we measured gaze position via eyetracking (for details, see below, Eyetracking). Whenever participants lost fixation (cutoff, 1.5° on horizontal axis) or blinked their eyes during these phases, the fixation mark would turn white and the data for the trial would be marked as faulty. Also, trials only commenced when participants’ gaze was at fixation. In total, participants performed 600 trials of this task without losing fixation. All trials were divided into two blocks of 300 trials, which in turn were subdivided into shorter blocks of 100 trials, in between which the participants could rest. After 300 trials on which fixation was not lost, the eyetracker was recalibrated (see below, Eyetracking).

#### Staircasing procedure

Participants performed a staircasing procedure during their intake session. After completing the staircasing procedure, task difficulty was fixed to be able to compare the effects of the pharmacological manipulation across all experimental sessions. The staircase task was almost identical to the primary task: stimulus properties, presentation time, and response window duration were the same. The ITI was prolonged to 450–650 ms. Participants received feedback on their performance on a trial-by-trial basis. The fixation dot turned green for correct answers and red for incorrect answers. An adaptation of the weighted up-down method proposed by Kaernbach (1991) was used to staircase performance at ∼75% correct, by changing the opacity of the Gabor patch. In short, corrections after incorrect responses were weighted differently than after correct responses, in a ratio of 3:1. The step size was 0.01 (opacity scale, 0–1, 1 is fully opaque); thus after errors, the opacity would be increased by 0.01 and after correct answers it would be decreased by 0.01/3. The procedure was aborted after 50 behavioral reversals, i.e., changes in sequences from correct to error or vice versa. The resulting difficulty level of the staircase procedure was calculated as the average opacity on reversal trials. In total, participants performed two blocks of this staircase procedure. The first block started at a high opacity (0.15), allowing the participants to familiarize with the stimulus. The second block started at the opacity obtained from the first block. The Gabor patch opacity level established during the second block of the staircasing procedure was used as the individuals’ difficulty level and fixed throughout the following three experimental sessions.

#### Localizer task

We included a localizer task in each experimental drug session, to be able to train decoding models on data in which behavioral responses were independent from the orientation of the Gabor. The localizer was similar to the discrimination task in terms of stimulus presentation times, response window duration, ITI, and stimulus characteristics (e.g., Gabor frequency and phase). During the localizer task, we centrally presented the same dynamic noise patches as during the discrimination task. On two-thirds of all trials, we additionally presented Gabor patches on full opacity that could be orientated CW/CCW. The Gabor patches were slightly tilted by 2°, rendering them more vertical (43° or −43°) or more horizontal (47° or −47°). Participants reported the orientation of Gabor patches as more horizontal by pressing “s” on the keyboard with the left index finger and as more vertical by pressing “k” with the right index finger. If only a dynamic noise patch was presented, participants were asked to refrain from responding. In total participants performed 840 trials, divided over two blocks of 420. The eyetracker was calibrated prior to block onset. Each block was subdivided in five miniblocks of 84 trials each, allowing participants to rest or close their eyes in between miniblocks (while keeping their head in the head-mount). Again, we excluded trials in which fixation was lost (>1.5° from fixation or during blinks) and replaced these excluded trials to get to a total of 840 trials.

### Data acquisition and preprocessing

#### Eyetracking

Gaze position and pupil size were measured with an EyeLink 1000 (SR Research) eyetracker during the experiment at 500 Hz. Gaze position was monitored to ensure participants’ gaze remained at or near fixation (within 1.5°, horizontal axis). Trials on which fixation was lost, due to shifts in gaze or blinks, in the time interval from stimulus onset to the end of the response window, were marked as bad. All bad trials were appended as new trials at the end of each block, ensuring that eventually 300 usable trials were performed in each of the two blocks of the main task. A nine-point calibration was used at the start of each block. To minimize movement of the participant, we used a head-mount with chinrest. Throughout the experiment, participants were instructed to move their heads as little as possible and to try to blink after they made their response. All pupil traces were low-pass filtered with a cutoff frequency of 10 Hz, blinks were linearly interpolated, and effects of blinks and saccades on pupil diameter were removed via deconvolution (Knapen et al., 2016). Then, within each block, pupil traces were normalized as the percentage difference from the block mean.

Next, we calculated the average pupil size for every trial in a short window (−500 to 0 ms) before the presentation of a Gabor stimulus. All trials were then subdivided into five equally populated bins based on the average prestimulus pupil size. Because ATX is known to increase pupil size (Nuiten et al., 2023), bins were created within each drug session and within each block of the experiment separately, to exclude the possibility that specific pupil bins contained more trials belonging to certain drug conditions or block numbers. By performing the binning analyses separately for the first and second block of the task, we also eliminated the effect of changes in pupil size between the first and second block that could potentially have stemmed from a (slightly) altered distance to the eyetracker following recalibration. Thus, pupil bins captured fluctuations in prestimulus pupil-linked arousal, irrespective of drug condition and block number.

#### EEG

EEG data were recorded with a 64-channel BioSemi apparatus (BioSemi B.V.), at 512 Hz. Vertical eye movements were recorded with electrodes located above and below the left eye, and horizontal eye movements were recorded with electrodes located at the outer canthi of the left and the right eye. The preprocessing pipeline for EEG data was the same for the discrimination and localizer tasks. All EEG traces were rereferenced to the average of two electrodes located on the left and right earlobes. The data were high-pass filtered offline, with a cutoff frequency of 0.01 Hz. Next, bad channels were detected automatically via a random sample consensus algorithm (RANSAC), implemented in the Autoreject Python package (Jas et al., 2017). This algorithm detects noisy channels on the basis of correlations between simulated data (derived from data of a subset of epochs, averaged across channels) and the actual data of EEG channels. Channels with a low correlation score were marked as noisy and subsequently interpolated via spline interpolation. Next, epochs were created by taking data from −2,000 to 2,000 ms around onset of stimulus presentation. To remove eyeblink artifacts, an independent component analysis (ICA; 25 components) was performed on the epoched data and components that strongly correlated to vertical EOG data were removed. On average 1.17 (SD: 0.40; maximum, 2) components were rejected per file in the discrimination task and 1.27 (SD: 0.50; maximum, 3) were rejected in the localizer task. The remaining artifacts were automatically detected by using the same RANSAC algorithm as before but on epoched data. Bad segments were repaired via interpolation if the artifactual data was present in only a few channels, but if more channels were affected the epoch was removed from the EEG data. On average 8.47% (SD: 7.30%, maximum: 47.15%) of all epochs were removed in the discrimination task and 8.47% (SD: 7.30%, maximum: 39.24%) in the localizer task. Lastly, the current scalp density (CSD) was computed using the surface Laplacian to attenuate the effects of volume conductance (Cohen, 2014).

### Data analysis

All behavioral analyses were programmed in Python 3.7. All trials that were marked due to lost fixation were disregarded from all analyses. EEG analyses were performed with the use of the Python package MNE (version 0.24.0; Gramfort et al., 2013). All trials were used for analysis of behavioral data, but only unrejected EEG epochs were included in EEG analyses. Note that all behavioral measures derived from the entire behavioral dataset were almost perfectly correlated to the behavioral measures derived from the subsample of data that was used for the EEG analysis (all *r* > 0.997).

#### Statistical analysis of physiological data, side effects, and self-reports

Heart rate and blood pressure were measured on three occasions during each session. We reported effects of ATX and DNP on pupil size, subjective measures of arousal, and occurrence of pharmacological side effects in Nuiten et al. (2023). In short, we observed that ATX robustly increased several measures of bodily arousal, such as pupil size, heart rate, and blood pressure, whereas DNP did not significantly modulate these measures. Although bodily effects of ATX were strong, subjective measures of alertness and calmness (as assessed with the visual analog scale; Bond and Lader, 1974) were unaffected (we also observed no effects of DNP).

#### Statistical analysis of behavioral data

Missed (no response) trials and trials with reaction times (RT) longer than 1,400 ms and shorter than 100 ms were excluded from behavioral and EEG analyses. To investigate effects of drug condition on perceptual and metacognitive performance, we used measures derived from signal detection theory (SDT; Green and Swets, 1966). We calculated perceptual sensitivity (*d*’) and bias (criterion) to assess perceptual performance. We estimated metacognitive sensitivity (meta-*d*’) and metacognitive bias (meta-criterion) using the maximum likelihood estimation approach of Maniscalco and Lau (2012). This approach takes perceptual performance into account when estimating metacognitive capacities, by comparing the sensitivity of metacognitive decisions of a participant to those of an ideal observer under SDT assumptions (Maniscalco and Lau, 2012). To establish the effects of drug condition on perceptual and metacognitive decision-making, we tested for drug effects on *d*’ and meta-*d*’, respectively. Moreover, we tested the interaction effect between drug condition and behavioral measure (*d*’ vs meta-*d*’) to establish whether the effect of drug condition on *d*’ was larger than on meta-*d*’. Although such a direct comparison of two experimental effects is necessary to establish whether a manipulation differentially affects two outcome measures, such direct tests are often overlooked (Nieuwenhuis et al., 2011; Meyen et al., 2022). To perform this interaction effect, we first calculated a measure of metacognitive efficiency referred to as *M*-diff (meta-*d*’ − *d*’) and then tested for drug effects. We furthermore decided to use *M*-diff as our primary measure of metacognitive efficiency rather than the other common measure—*M*-ratio (meta-*d*’ / *d*’), because for both pairs of comparisons (i.e., atomoxetine/donepezil vs placebo) the paired difference in *M*-ratio scores was not normally distributed and thus required a normalization correction (Shapiro–Wilk test, atomoxetine-placebo: *W *= 0.84, *p *< 0.001; donepezil-placebo: *W *= 0.91, *p *= 0.02). In contrast, the differences in *M*-diff were normally distributed for both pairs (Shapiro–Wilk test, atomoxetine-placebo: *W *= 0.97, *p *= 0.59; donepezil-placebo: *W *= 0.95, *p *= 0.23). The standard solution for normalizing a variable, i.e., a log-transform, could not be performed as there were three participants who had a negative *M*-ratio during one of their drug sessions. We did not want to interfere with our raw data too much and therefore decided not to apply several normalization steps (e.g., first adding a constant, then log-transforming) but rather use *M*-diff to quantify whether drug condition disproportionally affected *d*’ over meta-*d*’.

In a previous and similar study, we reported that atomoxetine increased *d*’ in the same group of participants during a highly similar visual discrimination task (Nuiten et al., 2023). We therefore had a clear prediction about the expected direction of the effect and thus tested the effect of ATX on *d*’ with a one-sided *t* test (*x̄* > 0). We used two-sided *t* tests to test the effects of drug condition on all other perceptual and metacognitive SDT measures. We used Bayesian equivalents of most statistical tests (Cauchy scale = 0.707) to quantify the evidence in favor of the null hypothesis. Note that all Bayes factors (BF) are reported as evidence in favor of the null hypothesis (BF_01_) and can be interpreted as anecdotal (1 < BF_01 _< 3), substantial (3 < BF_01 _< 10), strong (10 < BF_01 _< 30), and very strong (BF_01 _> 30; Jarosz and Wiley, 2014). Inversely, a BF_01 _< 1 should be interpreted as evidence for the alternative hypothesis following the formula:

$$BF_{01}=\frac{1}{BF_{10}}.$$

Thus, 0.10 < BF_01 _< 0.33 should be considered substantial evidence in favor of the alternative hypothesis, 0.03 < BF_01 _< 0.10 as strong evidence for the alternative hypothesis, etc.

We performed a secondary hierarchical Bayesian analysis to establish effects of drug condition on *M*-ratio. To this end, we used the HMeta-d toolbox (Fleming, 2017) implemented in R. The hierarchical Bayesian approach allows to estimate *M*-ratio at the group level, while taking single-subject level uncertainty into account. We fitted two classes of models. For the first class of models, we used the default function fit_metad_groupcorr.R to test for drug effects on *M*-ratio at the group level in a pairwise manner, meaning that we separately tested ATX versus PLC in one model and DNP versus PLC in another model. From these models, we obtained group-level posterior distributions of log-transformed *M*-ratio values for drug (ATX/DNP) and PLC. We tested for drug effects by subtracting the PLC posterior from the drug posterior and then quantifying the proportion of the distribution overlapping with 0, as well as calculating the 95% credibility interval for the difference distribution. The second class of models was used to establish a linear relation between prestimulus pupil size bin and *M*-ratio. We adapted default functions from the toolbox assuming a 2 × 2 repeated-measures design (fit_metad_2wayANOVA.R), to be able to fit a linear regression on *M*-ratio scores with pupil bins as regressor. The output from this model was a posterior distribution of beta-weights describing the linear relation between pupil bin and *M*-ratio. We analyzed this posterior distribution similarly to the difference posterior distribution from the drug models. The posterior distributions for log-transformed *M*-ratios (drug models) and beta-weights (pupil bin model) were estimated from 10.000 samples following 2,000 warm-up iterations, from three chains. Chain convergence was assessed with $\hat{R}$, which was <1.1 for all parameter estimates.

In a previous study (Nuiten et al., 2023), we investigated the effects of ATX and DNP on latent parameters underlying the decision-process, through drift diffusion modeling (DDM; Ratcliff and McKoon, 2008). Here, we did not use computational modeling of choice behavior, because regular DDMs are not well suited for tasks that have more than two response options (Ratcliff et al., 2016), as is the case in the current behavioral task (four response options: orientation × confidence). Previously, however, we observed that ATX increased drift rate, meaning that the rate at which sensory evidence was accumulated toward a decision threshold was increased, resulting in enhanced behavioral performance (Nuiten et al., 2023).

#### Pupillometry: polynomial regression

The analysis of pupillometry and the fitting procedure to behavioral measures of interest was similar to Beerendonk et al. (2024). To assess the relationship between prestimulus pupil-linked arousal and perceptual and metacognitive decision-making, we performed polynomial regressions. For every participant and pupil bin, we calculated mean decision confidence, *d*’, meta-*d*’ and *M*-diff. Next, we fitted first-order and second-order polynomial regression models to test whether prestimulus pupil size exhibited a, respectively, linear or quadratic relation with these behavioral measures. The regression formulae were defined as follows:

$$1)\;y\;∼\;\beta_{0}1+\beta_{1}P^{1}\;\mathrm{for}\;\mathrm{linear}\;\mathrm{regression},$$

$$2)\;y\;∼\;\beta_{0}1+\beta_{1}P^{1}+\;\beta_{2}P^{2}\;\mathrm{for}\;\mathrm{quadratic}\;\mathrm{regression},$$

with *y* being the respective behavioral measure (*d*’, proportion of high confident decisions, meta-*d*’ and *M*-diff), $\beta_{0}$ being the intercept, $\beta_{1}$ being the beta-weight of the first-order regressor, $\beta_{2}$ being the beta-weight of the second-order regressor, and *p* being the prestimulus pupil bin. We tested beta-values for significance with two-sided one-sample *t* tests against 0. Although the independent variable was always pupil bin number (1 through 5), we plot the regression curves against the mean prestimulus pupil size within each bin (as % of block mean) and not raw bin number.

#### EEG: ERP analysis

We tested the effects of drug condition, accuracy, confidence, and all interactions on the CPP and FP. For statistical analyses, epoched data were downsampled to 128 Hz. To examine response-locked evidence accumulation signals (i.e., CPP), we used electrodes CPz, Cp1, and Cp2 (O’Connell et al., 2012; Kelly and O’Connell, 2013; van Kempen et al., 2019; van Vugt et al., 2019; Nuiten et al., 2023), and we used electrodes Fz, F1, and F2 to examine frontal signals related to metacognitive decision-making (i.e., frontal potential, FP; Lim et al., 2020; Feuerriegel et al., 2022). To calculate ERPs, we first normalized epochs by subtracting the average baseline activity −80 to 0 ms before stimulus onset. Next, we averaged epochs within drug session, decision accuracy (correct/incorrect), and decision confidence (high/low). Because we previously established effects of ATX and DNP on CPP amplitude in the same participants during a similar task (Nuiten et al., 2023), we first performed pairwise one-sided *t* tests (ATX > PLC and DNP < PLC) to test for main effects of drug condition on CPP amplitude. Next, we performed cluster-corrected (cluster alpha = 0.05) pairwise (ATX/DNP vs PLC) 2 × 2 × 2 (drug × accuracy × confidence) rmANOVAs over time to establish main and interaction effects for each active drug versus PLC. Furthermore, we used cluster-corrected 3 × 2 × 2 (drug × accuracy × confidence) rmANOVAs to test the effects of accuracy, decision confidence, and their interaction, thereby leveraging the full factorial design (but ignoring omnibus main drug effects and interaction effects).

Lastly, we performed a set of regressions analyses (similar to above) in which we modeled the relation between CPP peak amplitude (Fig. 3*B*) and the interaction effect FP (accuracy × confidence; Fig. 3*D*) as the dependent variables versus prestimulus pupil bin as the independent variable.

#### EEG: multivariate pattern analysis

We investigated effects in terms of neural activity related to the processing sensory input by testing (1) if and how processing of sensory input was modulated by drug condition; (2) how sensory processing related to decision accuracy (correct vs incorrect responses), confidence (high vs low), and metacognitive accuracy (the interaction between confidence and performance); and (3) whether drug condition modulated how sensory processing was related to decision accuracy, decision confidence, and metacognitive accuracy. To test this, we performed multivariate pattern analyses (MVPA) on the EEG data, allowing us to inspect how the task-relevant stimulus dimension of the discrimination task—stimulus orientation—was represented in neural activity. Specifically, we trained a linear discriminant classifier (LDA) on an independent localizer task to discriminate CW versus CCW stimuli and tested the classifier on independent data from the main task. Because responses in the localizer task were independent to stimulus orientation in this task, the classifiers only extracted information related to stimulus orientation without being contaminated by motor preparation and execution. We additionally performed a 10-fold within-localizer decoding procedure, to investigate how Gabor orientation information was represented in neural data of the localizer task. Classification algorithms were trained and tested on the CSD-transformed EEG data from all 64 EEG electrodes. We used a temporal generalization decoding approach (generalization across time, GAT; King and Dehaene, 2014) in which classifiers were trained on each timepoint in the localizer and then tested on all timepoints in the localizer and in the main task, to test how activity patterns underlying stimulus orientation generalized over time. Classifier performance for all analyses was calculated as area under the curve (AUC). Trial counts for stimulus orientations and responses were balanced in the training dataset before the decoding procedure, by randomly under-sampling, concatenating over stimulus tilt and response accuracy (i.e., stimulus orientation and response were balanced in counts). The classifiers were tested on subsamples of the dataset, split up for drug condition, accuracy, and confidence (i.e., 3 × 2 × 2 factorial design). We averaged AUC scores across all these conditions to obtain GAT matrices of orientation decoding for every participant, which we tested for above-chance decoding with cluster-corrected one-sided (AUC > 0.5) *t* test. Next, we extracted AUC values from this cluster and tested for effects of drug condition, accuracy, confidence, and all interactions with rmANOVAs similar to the ERP analysis.

As raw classifier weights cannot be interpreted as neural sources of activity, spatial activity patterns from the localizer task were reconstructed from the decoder weights by multiplying the weights with the noise covariance in the EEG data, which creates topographic maps that can be meaningfully interpreted as neural effects (Haufe et al., 2014).

#### Data and code accessibility

Raw data and analysis scripts are publicly available and accessible at FigShare (https://doi.org/10.21942/uva.26235413).

## Results

We employed a within-subject, randomized, double-blind crossover design in which ATX (40 mg), DNP (5 mg), and a placebo (PLC) were administered in different EEG recording sessions, while drug order was counterbalanced between participants (Fig. 1*A*; see Materials and Methods). ATX and DNP, respectively, elevate catecholaminergic and cholinergic levels, as compared with PLC. As previously reported, ATX increased several measures of bodily arousal, including heart rate, blood pressure, and baseline pupil size compared with PLC, but DNP had no significant effects (data shown in Nuiten et al., 2023).

### Descriptive statistics of RT, *d*’, and meta-*d*’

Participants discriminated the orientation of Gabor patches as clockwise (CW, 45°) or counterclockwise (CCW, −45°), while simultaneously reporting the confidence (high or low) in their perceptual judgment (Fig. 1*B*). To assess perceptual and metacognitive effects, we calculated perceptual sensitivity (*d*’), the overall ratio of high confident answers, and metacognitive sensitivity (meta-*d*’) derived from (extensions of) signal detection theory (SDT; Green and Swets, 1966; Fleming and Lau, 2014; see Materials and Methods). Furthermore, we calculated *M*-diff (meta-*d*’ − *d*’) and *M*-ratio (meta-*d*’ / *d*’) to assess whether drug condition affected the absolute (*M*-diff) and relative (*M*-ratio) difference between perceptual and metacognitive sensitivity. We used *M*-diff to perform a direct test of the interaction effect between drug condition and sensitivity type, necessary for establishing whether drug effects on perceptual sensitivity were larger or smaller than on metacognitive sensitivity in absolute terms (see Materials and Methods for further substantiation of this decision; Nieuwenhuis et al., 2011; Meyen et al., 2022).

Across all pharmacological conditions, mean reaction times (RT) were 690 ms (SD = 108 ms), and mean perceptual sensitivity (*d*’) was 1.46 (SD = 0.48; corresponding to ∼75% correct, i.e., titrated level; see Materials and Methods). Participants had metacognitive insight in their performance, as meta-*d*’ was significantly above zero (*µ* meta-*d*’ = 0.75, SD = 0.40; one-sample *t* test against 0: *t*_(26)_ = 9.90, *p *< 0.001, *d *= 1.91). Overall, metacognitive evaluations were “inefficient,” however, because *M*-diff was significantly lower than 0 (*µ M*-diff = −0.70, SD = 0.50; one-sample *t* test against 0: *t*_(26)_ = −7.27, *p *< 0.001, *d *= 1.40), as observed more often in similar tasks (Hauser et al., 2017; Samaha and Postle, 2017; Shekhar and Rahnev, 2021a,b). A subzero *M*-diff is often interpreted as an indication that less sensory evidence was available for the metacognitive decision as compared with the perceptual decision (Fleming and Lau, 2014).

### Catecholaminergic enhancement improves perceptual, but not metacognitive decision-making

Previously, we established that ATX administration enhances *d*’ (no effect of DNP; Nuiten et al., 2023), and here we replicate this observation: ATX increased *d*’ compared with PLC, whereas DNP did not (ATX, one-sided *t* test: *t*_(26)_ = 2.06, *p *= 0.02, *d *= 0.39, BF_01 _= 0.40; DNP: *t*_(26)_ = 1.04, *p *= 0.31, *d *= 0.20, BF_01 _= 3.00; Fig. 1*C*). Drug condition did not significantly affect the ratio of high confidence answers (ATX: *t*_(26)_ = 0.49, *p *= 0.63, *d *= 0.09, BF_01 _= 4.39; DNP: *t*_(26)_ = 0.04, *p *= 0.97, *d *= 0.01, BF_01 _= 4.90; Fig. 1*D*). Contrary to the effects on *d*’, neither ATX nor DNP significantly modulated meta-*d*’ and Bayesian analyses provided moderate evidence for the absence of effects (ATX: *t*_(26) _=_ _−0.84, *p *= 0.41, *d *= 0.16, BF_01 _= 3.55; DNP: *t*_(26) _=_ _−0.46, *p *= 0.65, *d *= 0.07, BF_01 _= 4.44; Fig. 1*E*). Next, we further substantiated whether the effects of ATX on *d*’ (enhancement) and meta-*d*’ (no effect) indeed differed, by testing the interaction between drug condition (ATX/DNP vs PLC) and measurement (*d*’ vs meta-*d*’; Nieuwenhuis et al., 2011; Meyen et al., 2022). To do so, we calculated *M*-diff (meta-*d*’* *−* d*’) and show that it was lower for ATX versus PLC, but not for DNP versus PLC (ATX: *t*_(26) _=_ _−2.13, *p *= 0.04, *d *= 0.47, BF_01 _= 0.71; DNP: *t*_(26) _=_ _−1.21, *p *= 0.24, *d *= 0.23, BF_01 _= 2.54; Fig. 1*F*). Thus, ATX specifically improved perceptual decision-making (*d*’) without affecting metacognitive decision-making (meta-*d*’) and significantly increased the absolute difference between meta-*d*’ and *d*’ (*M*-diff), showing that ATX disproportionally increased perceptual over metacognitive decision-making.

Additionally, we analyzed whether the ratio between *d*’ and meta-*d*’, reflected in the measure *M*-ratio (meta-*d*’ / *d*’) was altered by our pharmacological manipulation. We used a hierarchical Bayesian approach to obtain posterior distributions of log-transformed *M*-ratio estimates, for each of the three drug conditions (see Materials and Methods). We calculated the difference between the posterior distribution of the active drug condition (ATX and DNP) and the posterior distribution of PLC (Fig. 1*G*). Although for both ATX and DNP, the mean difference in *M*-ratio with PLC was below zero, indicative of a lowered *M*-ratio, the 95% credibility intervals (CI_95%_) overlapped with zero suggesting that *M*-ratio was likely not affected by either drug (difference ATX-PLC log(*M*-ratio), *μ* = −0.25, SD = 0.16, CI_95% _=_ _[−0.59, 0.07]; difference DNP-PLC log(*M*-ratio), *μ* = −0.09, SD = 0.13, 95% CI_95% _=_ _[−0.36, 0.17]). Indeed for both drugs, the probability that the drug effect was larger than 0 was larger than 0.05, suggesting that neither drug modulated *M*-ratio [ATX: *p*(difference > 0) = 0.06; DNP: *p*(difference > 0) = 0.23; Fig. 1*G*].

Due to repeated exposure to the task, and fixed task difficulty across drug sessions, *d*’ significantly increased over the course of the three sessions (one-factor repeated-measures ANOVA; *F*_(2,52)_ = 10.97; *p *< 0.001; *η*_p_^2 ^= 0.30; BF_01 _= 0.01). Training in the behavioral task did not, however, result in a change in meta-*d*’ (one-factor repeated-measures ANOVA; *F*_(2,52)_ = 1.30; *p* = 0.28; *η_p_*^2 ^= 0.05; BF_01 _= 3.43). Although the order of drug administration was counterbalanced between individuals, we wanted to exclude the possibility that the main effects of ATX on *d*’ were driven by training effects. To do so, we performed a mixed-level ANOVA, in which we included drug condition as the within-subject variable and drug order (consisting of six levels, given there were three drugs) as between-subject variable. We only performed this analysis for ATX (vs PLC), as DNP did not influence *d*’ whatsoever. The mixed-level ANOVA revealed no main effect of drug order (*F*_(5,21)_ = 0.74; *p* = 0.60; *η_p_*^2 ^= 0.15; BF_01 _= 4.18) but did show a main effect of drug condition (*F*_(1,21)_ = 4.66; *p* = 0.04; *η_p_*^2 ^= 0.18; BF_01 _= 0.69). Crucially, there was no interaction between drug order and drug condition (*F*_(5,21)_ = 1.50; *p* = 0.23; *η_p_*^2 ^= 0.26; BF_01 _= 0.84). Therefore, it is unlikely that the effect of ATX on *d*’ was driven by behavioral training of the task.

Neither ATX nor DNP significantly affected reaction times (RT; ATX: *t*_(26)_ = 0.82, *p *= 0.42, *d *= 0.09, BF_01 _= 3.62; DNP: *t*_(26)_ = 1.00, *p *= 0.33, *d *= 0.13, BF_01 _= 3.13), response criterion (c; ATX: *t*_(26)_ = 0.12, *p *= 0.91, *d *= 0.02, BF_01 _= 4.88; DNP: *t*_(26)_ = 0.45, *p *= 0.66, *d *= 0.08, BF_01 _= 4.48), and metacognitive bias (meta-c; ATX: *t*_(26)_ = 0.31, *p *= 0.76, *d *= 0.05, BF_01 _= 4.69; DNP: *t*_(26)_ = 0.05, *p *= 0.96, *d *= 0.00, BF_01 _= 4.90) as compared with PLC.

Summarizing, ATX enhanced perceptual sensitivity (*d*’) but did not significantly affect metacognitive sensitivity (meta-*d*’). Moreover, ATX significantly lowered *M*-diff, demonstrating that it disproportionally increased the absolute difference between perceptual sensitivity and metacognitive sensitivity. Although the direction of the ATX effect on *M*-ratio was in line with its effect on *M*-diff, the 95% credibility interval for this effect overlapped with zero suggesting the absence of an effect of ATX on *M*-ratio. Elevated cholinergic levels did not robustly modulate behavior in any way.

### Prestimulus pupil-linked arousal partially mimics ATX effects

Thus, the pharmacological elevation of catecholaminergic activity (through ATX) affected perceptual sensitivity in the absence of metacognitive sensitivity effects. To further explore the reliability and generality of these effects, we next focused on a measure thought to reflect spontaneous or task-related fluctuations in, among others, catecholaminergic (but also cholinergic) activity, namely, pupil size (Reimer et al., 2014; McGinley et al., 2015; Joshi et al., 2016). Fluctuations in prestimulus pupil-linked arousal have previously been shown to modulate *d*’ during perceptual decision-making tasks (McGinley et al., 2015; Waschke et al., 2019; Podvalny et al., 2021; Beerendonk et al., 2024), but its relation to meta-*d*’ and *M*-diff has not been established. To assess the relation between fluctuations in pupil-linked arousal on our measures of task performance, we subdivided all trials into five equally sized bins based on the average pupil size in a short window (−500 to 0 ms) before the presentation of each Gabor stimulus (similar to Beerendonk et al., 2024; see Materials and Methods). We performed the binning procedure within each drug session and for each of the two experimental blocks separately. This approach ensures that each of the five pupil bins contains the same number of trials from each drug condition and experimental block, to exclude the possibility that pupil bin number would be driven by pharmacological effects or slow drifts in pupil size. Next, *d*’, overall confidence rate, meta-*d*’, and *M*-diff were calculated for every participant and pupil bin. Because the relationship between pupil size and choice behavior has been shown to be inverted u-shaped as well as linear, first- and second-order polynomial regressions were fitted (see Materials and Methods).

Replicating previous work (McGinley et al., 2015; Beerendonk et al., 2024), and in line with the behavioral results associated with our drug manipulation, *d*’ exhibited a clear relationship with prestimulus pupil diameter (Fig. 1*H*, top left panel). Specifically, both first-order (*β*_1_) and second-order (*β*_2_) fits were significant showing that *d*’ increased with pupil diameter for low to medium levels of pupil-linked arousal but was lowered at the extremes of high arousal (*β*_1_: *t*_(26)_ = 3.79, *p *< 0.001, *d *= 0.73, BF_01 _= 0.02; *β*_2_: *t*_(26)_ = −3.79, *p *< 0.001, *d *= 0.73, BF_01 _= 0.02). This increase in *d*’ was matched in decision confidence, which was also enhanced with elevated levels of pupil-linked arousal (*β*_1_: *t*_(26)_ = 2.89, *p *= 0.01, *d *= 0.56, BF_01 _= 0.17; *β*_2_: *t*_(26)_ = −2.66, *p *= 0.01, *d *= 0.51, BF_01 _= 0.27; Fig. 1*H*, top right panel). Prestimulus pupil-linked arousal was not related to meta-*d*’ (*β*_1_: *t*_(26)_ = 0.56, *p *= 0.58, *d *= 0.11, BF_01 _= 4.26; *β*_2_: *t*_(26)_ = −1.00, *p *= 0.33, *d *= 0.19, BF_01 _= 3.13; Fig. 1*H*, bottom left panel). Crucially, baseline pupil-linked arousal exhibited a negative linear relation with *M*-diff, indicating that *d*’ and meta-*d*’ were increasingly disproportionally affected for higher levels of pupil-linked arousal (*β*_1_: *t*_(26)_ = −3.12, *p *= 0.004, *d *= 0.60, BF_01 _= 0.11; *β*_2_: *t*_(26)_ = 1.07, *p *= 0.29, *d *= 0.21, BF_01 _= 2.92; Fig. 1*H*, bottom right panel). Finally, we tested for a similar linear relation between prestimulus pupil bin and *M*-ratio by fitting a hierarchical Bayesian model (see Materials and Methods). Although the mean of the posterior distribution fell below zero (*μ *=* *−0.02, SD = 0.03; in line with a negative linear relation between pupil bin and *M*-ratio), the 95% CI of the posterior overlapped with 0 (CI_95%_ = [−0.08, 0.04]; Fig. 1*I*).

Thus, similar to pharmacological elevations of catecholaminergic activity, elevated prestimulus pupil size—indicative of global arousal changes and driven by among other catecholamine activity—was associated with changes in perceptual sensitivity but not metacognitive sensitivity. Furthermore, the absolute, but not relative, difference between perceptual and metacognitive sensitivity increased with elevated prestimulus pupil size.

### Catecholamines do not result in sharpened encoding of visual information

Next we employed the EEG data to investigate whether electrophysiological responses were modulated by drug, decision accuracy, and confidence. Note that neural effects related to metacognitive insight should manifest itself, if present, in the interaction between accuracy and confidence. Specifically, metacognitively correct answers are defined as high confidence correct and low confidence incorrect decisions, whereas metacognitively incorrect answers are defined as low confidence correct and high confidence incorrect decisions (Fleming and Lau, 2014).

**Figure 2.**
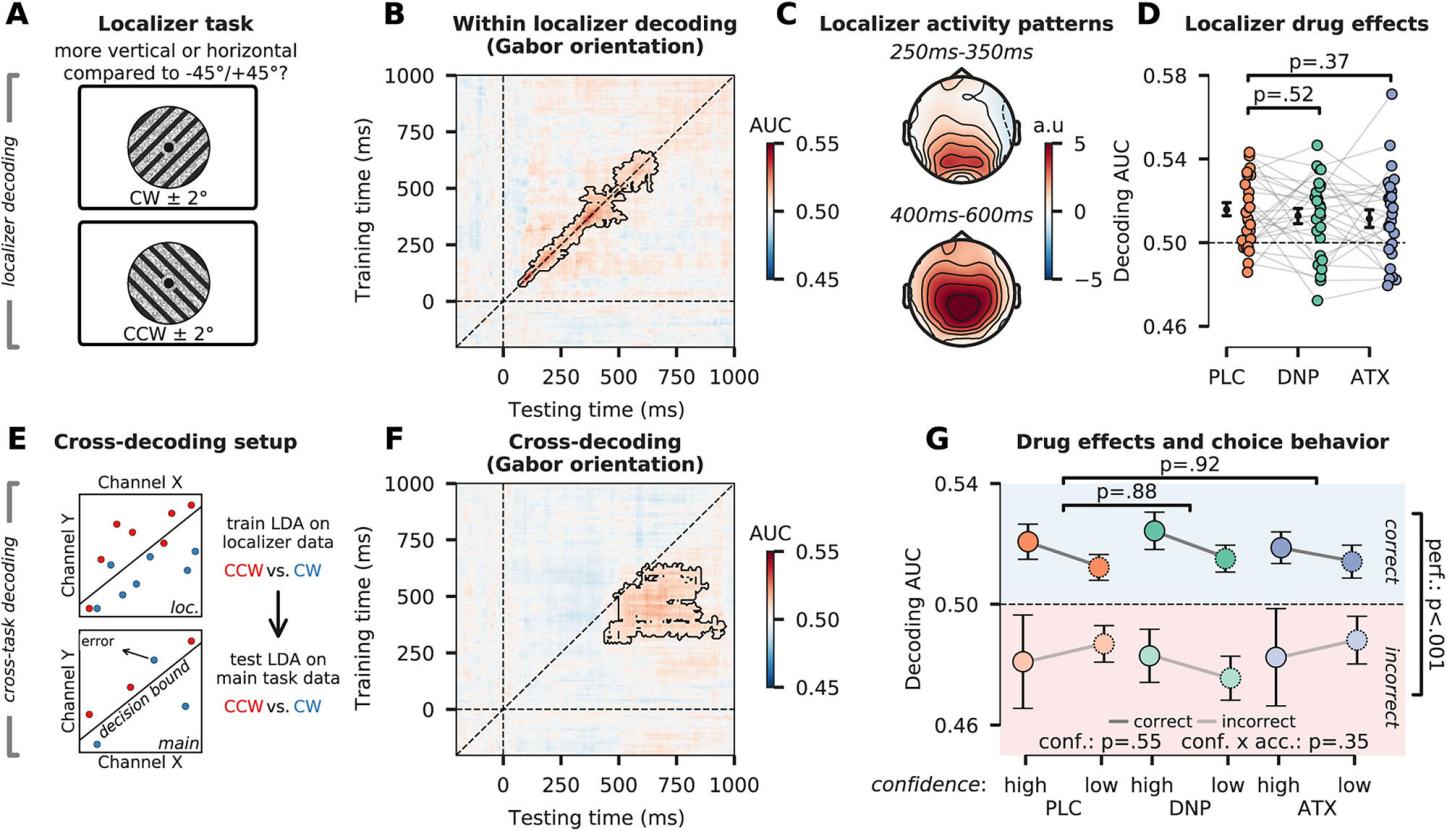
Multivariate decoding of Gabor orientation. ***A***, During the localizer task (illustration shown), participants reported a slight tilt offset of ±2° from diagonal, by pressing a button with their left hand (for horizontally tilted Gabors: −47° and +47°) or right hand (for vertically tilted Gabors: −43° and +43°). Gabor orientation and behavioral responses were therefore independent (see Materials and Methods for details). ***B***, Temporal generalization of stimulus orientation decoding in the localizer task (AUC). Training time is shown on the *y*-axis, testing time on the *x*-axis. Black demarked areas represent clusters that survived cluster-based permutation testing at *p* ≤ 0.05. ***C***, Topographic maps showing the activity patterns (from transformed classifier weights) in two separate time windows. ***D***, Tenfold within-localizer decoding AUC taken from the cluster of panel ***B***, separated by drug. ***E***, Schematic of cross-task decoding setup. A linear discriminant was trained to distinguish CCW (red dots, example data) from CW (blue dots) during the localizer task (top panel). This linear discriminant was then presented with data from the main task, after which it classified trials as containing CCW or CW information (bottom panel). ***F***, Temporal generalization of stimulus orientation cross-decoding (AUC) when training on the (localizer task, *y*-axis) and testing on the main task (discrimination task, on the *x*-axis). Black demarked areas represent clusters that survived cluster-based permutation testing at *p* ≤ 0.05. ***G***, Cross-decoding AUC taken from the cluster of panel ***E***, separated by drug, accuracy, and confidence. Note that conf. demarks the factor decision confidence and acc. demarks the factor decision accuracy. All error bars indicate SEM.

We performed MVPA to test how the encoding of the task-relevant visual stimulus feature (Gabor orientation) was affected by drug administration and how it related to decision accuracy, confidence, and metacognition. To this end, we trained a linear discriminant classifier (LDA) on data from an independent task (referred to as the localizer task) to discriminate CW versus CCW stimuli. By training the classifier on this independent dataset, we ensured that it was not being contaminated by motor preparation/execution (see Materials and Methods; Fig. 2*A*). During this localizer task, Gabor patches were presented at a slight tilt (−2° or +2°) rendering their orientation as being closer to the vertical axis (−43° or 43°) or the horizontal axis (−47° or 47°). Participants reported Gabor orientation as being closer to the vertical or the horizontal axis via button presses (see Materials and Methods). Note that during the localizer task, Gabor patches were presented at full opacity.

We first quantified how Gabor orientation was represented in neural data of the localizer task by performing a 10-fold within-localizer decoding procedure using a temporal generalization approach (GAT; Fig. 2*B*). Orientation representations in neural activity were observed on the diagonal of the GAT matrix from 70 to 640 ms poststimulus, in line with previous studies (Fig. 2*B*; King et al., 2016). There was limited off-diagonal decoding, suggesting that orientation representations were “chained” and constantly changed over time during the localizer task (King and Dehaene, 2014). Such rapidly changing neural representations have previously been observed for response-irrelevant stimulus features, such as Gabor orientation in the localizer task (Mostert et al., 2016; King and Wyart, 2021). Activation patterns were retrieved from decoding weights (as raw classifier weights are not directly interpretable as reflecting neural activations; Haufe et al., 2014) and showed that orientation information was initially present over occipital electrodes and was later most prominent over centroparietal ones (Fig. 2*C*). Drug condition did not modulate decoding accuracies within the cluster reported in Figure 2*B*, and Bayes factors revealed moderate evidence for an absence of any drug effect (ATX: *t*_(26)_ = −0.91, *p *= 0.37, *d *= 0.22, BF_01 _= 3.37; DNP: *t*_(26)_ = −0.66, *p *= 0.52, *d *= 0.16, BF_01 _= 4.03; Fig. 2*D*).

After verifying that (task-irrelevant) Gabor orientation information could be decoded from neural data of the localizer task, we next performed a cross-task decoding analysis in which classifiers that were trained on the localizer and tested on all timepoints of the main task (for more details see Materials and Methods). We first tested the classifier on all data from the discrimination task (irrespective of decision accuracy and confidence) to test whether we could cross-decode Gabor orientation (Fig. 2*E*). When averaging across drug sessions, we observed that stimulus orientation could be cross-decoded in a cluster from ∼430 to 945 ms (*p *= 0.002, cluster-corrected; Fig. 2*F*). The observed cluster was slightly off-diagonal, suggesting that there was a delay in the times when orientation information was initially decodable from the localizer task as compared with the discrimination task. This is likely because Gabor stimuli were presented at full contrast during the localizer task and at low contrast in the main task and increased stimulus contrast is known to speed up the latency of visually evoked responses (Groen et al., 2022). Neural representations generalized more over time when compared with the within-localizer decoding, possibly because orientation information during the main task was directly response relevant and thus needed to be sustained over time (King and Dehaene, 2014).

Next, we tested how drug condition, decision accuracy, decision confidence, and their interactions were related to sensory representations of Gabor orientation. To do so, we tested the classifier on EEG data from the discrimination task in a 3 × 2 × 2 (drug × accuracy × confidence) factorial design; i.e., the classifier was tested separately on the data of each of the 12 subconditions. This procedure effectively treats decision accuracy and confidence as independent variables, similar to the pharmacological manipulation. Although it is generally inappropriate to combine causal and correlational effects, it is necessary when investigating how neural processes leading up to specific behavioral outputs (e.g., errors) are modulated by an independent variable (e.g., pharmacological manipulation). Indeed, such approaches are common in research investigating the neural mechanisms underlying, for example, error processing, e.g., by testing causal effects on the error-related negativity (ERN) component (Tieges et al., 2004; Zirnheld et al., 2004; Barnes et al., 2014).

After testing the classifier on data from the main task, we extracted AUC values from the cluster reported in Figure 2*F* for every participant and subcondition and then performed a 3 × 2 × 2 (drug × accuracy × confidence) factorial rmANOVA to establish effects of accuracy, decision confidence and their interaction on classifier AUC (ignoring omnibus drug effects, while leveraging the full data design), and pairwise 2 × 2 × 2 (drug × accuracy × confidence) factorial rmANOVAs to test main and interaction effects of ATX and DNP versus PLC. We observed increased decoding AUC for correct versus incorrect answers (*F*_(1,26)_ = 26.85; *p *< 0.001; *η_p_*^2 ^= 0.51; BF_01 _= 1.14 × 10^−3^; Fig. 2*G*), but decision confidence (*F*_(1,26)_ = 0.36; *p *= 0.55, *η_p_*^2 ^= 0.01; BF_01 _= 5.13; Fig. 2*G*) and metacognitive accuracy were not associated with changes in AUC (interaction accuracy × confidence: *F*_(1,26)_ = 0.89; *p *= 0.35; *η_p_*^2 ^= 0.03; BF_01 _= 3.39; Fig. 2*G*). In line with previous work (Alilović et al., 2023), decoding AUC was significantly below chance level for incorrect decisions (one-sample, two-sided *t* test against 0.50: *t*_(26)_ = −4.34; *p *< 0.001; *d *= 0.83; BF_01 _= 6.83 × 10^−3^), suggesting that neural activity of incorrect trials contained “illusory” stimulus orientation information, i.e., information about the nonpresented stimulus orientation that was reported. Neither ATX nor DNP modulated overall decoding accuracy (ATX: *F*_(1,26)_ = 0.01, *p *= 0.92, *η*_p_^2 ^= 0.00, BF_01 _= 4.91; DNP: *F*_(1,26)_ = 0.02, *p *= 0.88, *η*_p_^2 ^= 0.00, BF_01 _= 4.94). Furthermore, neither drug significantly modulated how decision accuracy, decision confidence, and metacognitive accuracy (three-way interaction) were reflected in classification AUC (all *p *> 0.48).

Summarizing, although Gabor orientation could be reliably decoded from posterior and central electrodes, drug condition did not affect orientation decoding in any way, neither in the localizer task nor in the main task, and there were no interactions with decision accuracy and confidence in the latter. This suggests that elevated cholinergic and catecholaminergic levels do not modulate the encoding of task-relevant visual stimulus features and that the behavioral effects of catecholaminergic enhancement (on *d*’ and *M*-diff) were likely not related to changes in the encoding of task-relevant stimulus features.

### Catecholaminergic modulations of centroparietal evidence accumulation signals

The process of perceptual decision-making is thought to (sequentially) rely on the extraction of sensory information from stimulus input (reported above) followed by the integration of that sensory information into a (unsigned, i.e., not containing stimulus category information) decision variable that accumulates over time until a certain internal decision threshold is crossed (Gold and Shadlen, 2007; Ratcliff and McKoon, 2008; Shadlen and Kiani, 2013; Msheik et al., 2022). Previous primate work has shown that the parietal cortex is key for integrating sensory information into this decision variable (Gold and Shadlen, 2007; Kiani and Shadlen, 2009) and in humans, the CPP has been identified as an ERP component thought to reflect sensory evidence accumulation (O’Connell et al., 2012; Kelly and O’Connell, 2013; Loughnane et al., 2016; van Kempen et al., 2019; van Vugt et al., 2019; Nuiten et al., 2023). The CPP scales with RT, decision accuracy, and decision confidence (O’Connell et al., 2012; Kelly and O’Connell, 2013; van Vugt et al., 2019; Nuiten et al., 2023), and its peak amplitude has been shown to increase with elevated levels of catecholamines but decrease with elevated levels of acetylcholine (Loughnane et al., 2019; Nuiten et al., 2023).

**Figure 3.**
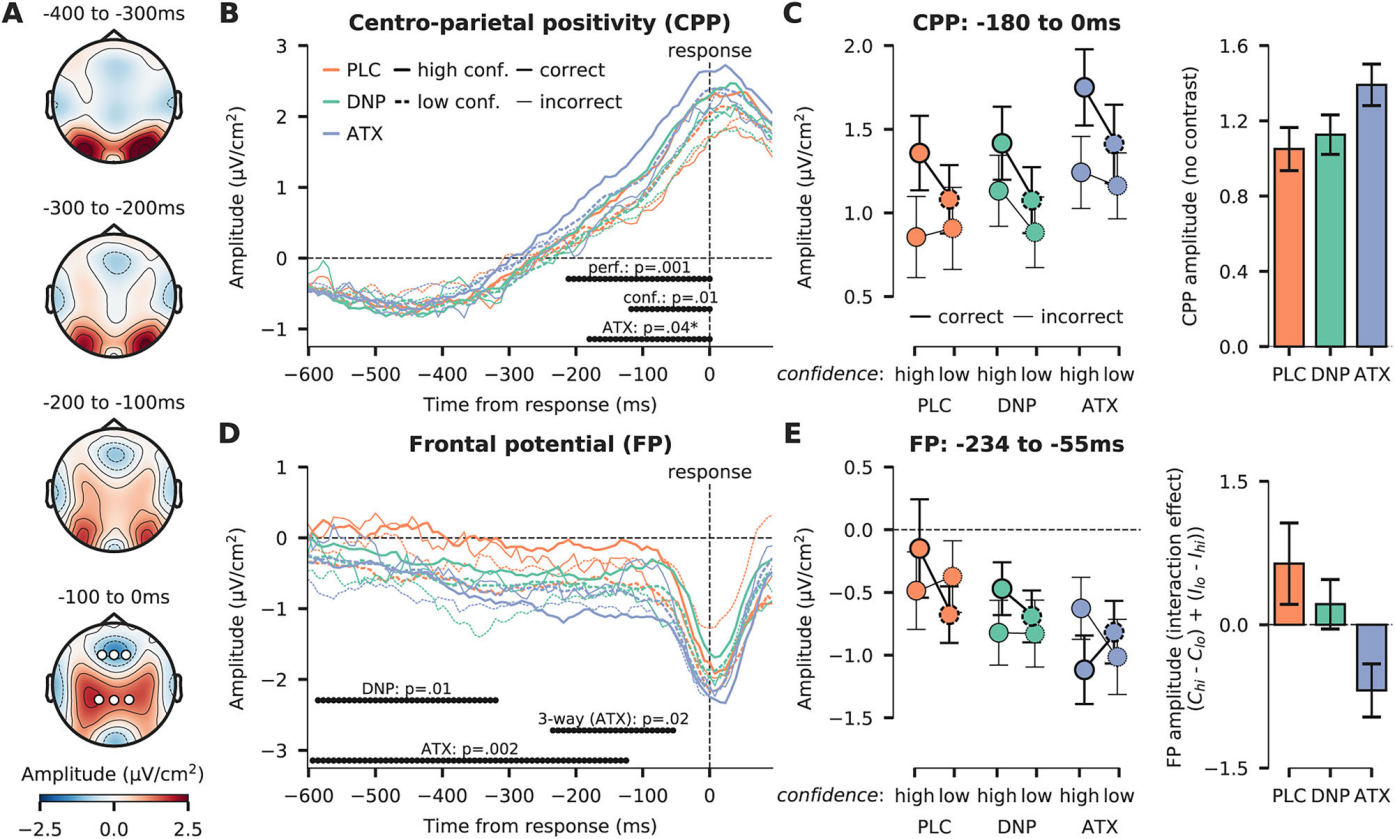
Response-locked ERPs over centroparietal and frontal cortical regions. ***A***, Topographic maps of response-locked activity, in steps of 100 ms. The bottom topographic map shows spatial ROIs for the CPP (three electrodes at over centroparietal areas: CP1, CP2, CPz) and the FP (three electrodes over frontal areas: F1, F2, Fz) analyses as white markers. ***B***, Response-locked CPP shown for the ATX, DNP, and PLC condition, split up for high confident (solid lines) versus low confident (dotted lines) and correct (opaque lines) versus incorrect (transparent lines) trials. The horizontally plotted black dots indicate effects that survived across time cluster-correction. *The effects of ATX were tested with a cluster-corrected one-sided *t* test (ATX > PLC, see Materials and Methods). ***C***, Average CPP amplitudes from the cluster in which ATX modulated CPP amplitude. The three drug conditions are plotted on the *x*-axis, separated for high and low confidence decisions. Correct trials are plotted in opaque lines and incorrect trials in transparent lines. The inset bar plot on the right shows the overall CPP amplitude (averaged across confidence and accuracy) for each drug. ***D***, Same as ***B***, but for FP ERP component. ***E***, Average FP amplitudes within the cluster where the three-way interaction (drug × accuracy × confidence) was significant. The three drug conditions are plotted on the *x*-axis, separated for high and low confidence decisions. Correct trials are plotted in opaque lines and incorrect trials in transparent lines. The inset bar plot on the right shows the FP amplitude interaction effect (accuracy × confidence) for each drug, defined as [(*C*_hi_ − *C*_lo_) + (*I*_lo_ − *I*_hi_)], with *C*_hi_, correct decision, high confidence; *C*_lo_, correct decision, low confidence; *I*_lo_, incorrect decision, low confidence; and *I*_hi_, incorrect decision, high confidence. All error bars indicate SEM.

In Figure 3*B*, we plotted the response-locked CPP for ATX, DNP, and PLC, for high versus low confidence trials and correct versus incorrect trials average across three electrodes over centroparietal regions [see Fig. 3*A*, bottom topomap for spatial region of interest (ROI) used for this analysis]. Electrode selection was identical to our previous study (Nuiten et al., 2023) and in line with other work in this field (O’Connell et al., 2012; Kelly and O’Connell, 2013; Loughnane et al., 2016; van Kempen et al., 2019). We replicated our previous finding that ATX increased CPP amplitudes, here in a time window from −180 to 0 ms preceding the response (cluster-corrected *p *= 0.04; Fig. 3*B*). Moreover, we observed increased CPP amplitudes for correct versus incorrect trials (cluster-corrected *p *= 0.001; −211 to 0 ms; Fig. 3*B*) and high confident versus low confident trials (cluster-corrected *p *= 0.01; −117 to 0 ms; Fig. 3*B*). None of the other effects survived cluster-correction or did we observe significant effects before −600 ms preresponse. In Figure 3*C*, we plot the mean CPP amplitudes for the full 3 × 2 × 2 design, averaged across the temporal cluster in which the main effect of ATX was significant (−180 to 0 ms). In sum, we found that ATX, decision accuracy, and confidence were all associated with increased CPP amplitude, but metacognitive accuracy was not reflected in the CPP (also not in interaction with ATX). ATX thus mainly increased the overall amplitude of the CPP but did not modulate the relationship between accuracy (correct/incorrect) and confidence (high/low). We did not replicate our previous observation that DNP attenuates the peak of the CPP (Nuiten et al., 2023).

### Catecholaminergic modulations of frontal EEG signals reflecting metacognitive insight

Recent fMRI studies have revealed that the computation of metacognitive insight during task performance is associated with activity in human prefrontal cortex (PFC) and causal interference of the anterior part of this region using TMS confirmed its crucial role in metacognition (Fleming et al., 2012b; D. Bang and Fleming, 2018; Morales et al., 2018; Lapate et al., 2020). Furthermore, human electrophysiology has revealed a (response-locked) ERP component averaged over frontal electrodes (F1, F2, Fz; see Fig. 3*A*, bottom row for spatial ROI), referred to as the frontal potential (FP; Lim et al., 2020; Feuerriegel et al., 2022), which has been shown to reflect upcoming decision confidence (Lim et al., 2020), but only for correct trials (Feuerriegel et al., 2022). This suggests that it may reflect processes associated with computing metacognitive evaluations of task performance.

In Figure 3*D* we plot the response-locked FP for all 12 conditions for the frontal ROI. Electrode selection was based on previous studies focusing on the FP component (Lim et al., 2020; Feuerriegel et al., 2022). In general, the FP component showed a gradually declining profile toward response execution, becoming more negative over time. Interestingly, we observed that ATX decreased FP amplitude even further as compared with PLC from −594 to −125 ms before the button press (cluster-corrected *p *= 0.002; Fig. 3*D*). DNP did similarly, but for a shorter time period (−586 to −320 ms, cluster-corrected *p *= 0.01). More importantly, we observed a three-way interaction between ATX (vs PLC), decision accuracy, and confidence right before response execution (from −234 to −55 ms, cluster-corrected *p *= 0.02; Fig. 3*D*). In Figure 3*E*, we plot FP amplitudes per drug condition separately to further reveal its profile. As can be observed, in the PLC and DNP condition, the amplitude of the FP clearly shows the interactive pattern indicative that FP tracks metacognitive accuracy. Specifically, the FP was least negative for correct answers given with high confidence, whereas FP amplitude decreased for correct answers with low confidence, replicating previous work (Feuerriegel et al., 2022). For incorrect answers, the confidence modulation was more subtle. Metacognitively correct answers (low confidence incorrect answers) were associated with the least negative FP (especially in the PLC condition), whereas metacognitively incorrect answers (high confidence incorrect answers) were associated with a relatively more negative FP. This is in line with the idea that the FP reflects metacognitive insight, most prominently in correct trials (Lim et al., 2020; Feuerriegel et al., 2022). However, remarkably, the ordering of these four conditions changed drastically when ATX was administered. To further quantify the effect of ATX, we calculated the overall “metacognitive effect” for each drug (Fig. 3*E*, right panel), reflecting the difference in FP amplitude between metacognitively correct answers and metacognitively incorrect answers ([high confidence correct − low confidence correct trials] + [low confidence incorrect − high confidence incorrect trials]). This measure can be interpreted as a reflection of overall metacognitive performance in neural activity. We observed that ATX inverted the magnitude of this effect entirely compared with PLC. This condition clearly indicates a lack of association between the FP and metacognitive insight, in line with the idea that ATX impoverishes metacognitive insight, compared with perceptual task performance (Fig. 1). In the Discussion, we will further elaborate on this finding.

### Pupil-linked arousal is related to a metacognitive signature in the FP

**Figure 4.**
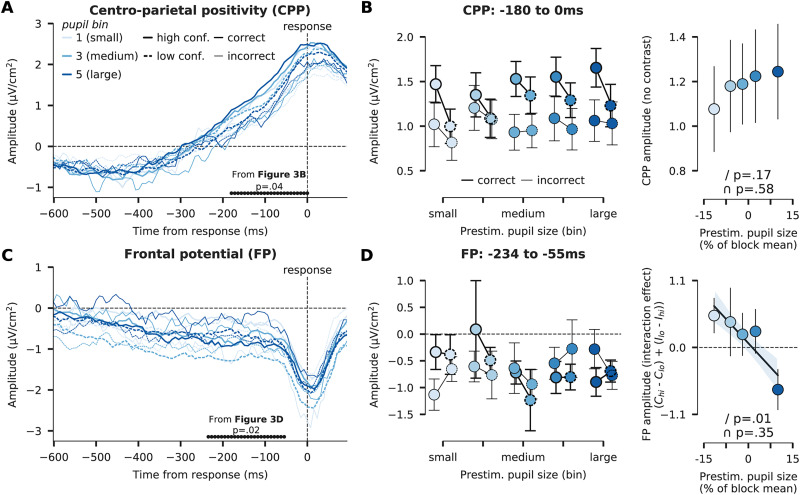
Relation between pupil size and the CPP and FP. Same as Figure 3, but now using five prestimulus pupil size bins as variables instead of the pharmacological manipulation. ***A***, Response-locked CPP shown for each pupil size bin, split up for high confident (solid lines) versus low confident (dotted lines) and correct (opaque lines) versus incorrect (transparent lines) trials. ***B***, CPP amplitudes extracted from the temporal cluster in ***A*** (statistics and cluster from Fig. 3*B*), shown for each condition as a factor of pupil size. The right panel shows the CPP amplitude per pupil bin and the regression results. ***C***, Response-locked FP (accuracy × confidence), as a factor of pupil size bin. ***D***, FP amplitudes extracted from cluster in ***C*** (same as in Fig. 3*D*), shown for each condition. The inset bar plot on the right shows the FP amplitude interaction effect (accuracy × confidence) for each drug, defined as [(*C*_hi_ − *C*_lo_) + (*I*_lo_ − *I*_hi_)], with *C*_hi_, correct decision, high confidence; *C*_lo_, correct decision, low confidence, high confidence; *I*_lo_, incorrect decision, low confidence; and *I*_hi_, incorrect decision. Note that the regression analyses of the utmost right panels were performed with bin number as independent variable, but the *x*-axis shows the mean pupil size (in % of block mean) for each bin. All error bars indicate SEM.

In a final analysis, similar to Figure 1, we used prestimulus pupil size as an indirect index of neuromodulator activity, rather than the pharmacological manipulation, and tested its relation to the univariate EEG effects. In Figure 4, we plot the CPP and FP for each of the five pupil bins, separated by decision accuracy and confidence. We calculated the mean CPP and FP amplitudes in the same time windows as reported in Figure 3*C* (−180 to 0 ms) and Figure 3*E* (−234 to −55 ms), but now per pupil bin instead of drug condition. For the CPP, we tested the presence of a linear relation with pupil bin, to directly link this analysis to the observed main effect of ATX on the CPP (Fig. 3*C*). Regression analysis did not reveal a significant relation between CPP amplitude and prestimulus pupil size, although numerically the effect went in the expected direction (*β*_1_: *t*_(26)_ = 1.41, *p *= 0.17, *d *= 0.27, BF_01 _= 2.02; *β*_2_: *t*_(26)_ = −0.57, *p *= 0.58, *d *= 0.11, BF_01 _= 4.24; Fig. 4*B*). Next, we extracted FP amplitudes from the time window in which we observed the cluster-corrected three-way interaction effect (ATX × accuracy × confidence; Fig. 3*D*). Then, we calculated the average interaction effect between decision accuracy and confidence (similar to above) within these timepoints and tested its relation with prestimulus pupil size. Regression analysis revealed that the metacognitive signature in the FP (explained above) became more negative with increasing prestimulus pupil size (*β*_1_: *t*_(26)_ = 2.62, *p *= 0.01, *d *= 0.51, BF_01 _= 0.29; *β*_2_: *t*_(26)_ = −0.95, *p *= 0.35, *d *= 0.18, BF_01 _= 3.27; Fig. 4*D*), suggesting that metacognitive accuracy, as reflected in FP, deteriorated when going from states of low versus high pupil-linked arousal.

## Discussion

Previous studies raised the suggestion that alterations in baseline neuromodulator levels may oppositely affect perceptual and metacognitive decision-making (Hauser et al., 2017; Gelbard-Sagiv et al., 2018; Nuiten et al., 2023). Here, we causally tested this hypothesis by pharmacologically elevating catecholamine (through atomoxetine, ATX) and acetylcholine (through donepezil, DNP) levels in human participants and testing the effects of these neuromodulator systems on perceptual and metacognitive decision-making behavior and associated neural activity (Green and Swets, 1966; Maniscalco and Lau, 2012; Fleming and Lau, 2014).

### Elevated catecholamine levels disproportionally increase perceptual over metacognitive sensitivity

Replicating and extending previous work (Hauser et al., 2017; Gelbard-Sagiv et al., 2018; Nuiten et al., 2023), we showed that elevated catecholaminergic levels improved perceptual (*d*’) but not metacognitive sensitivity (meta-*d*’; cholinergic effects were not robust; Fig. 1*C*,*E*). Crucially, the effects of ATX on *d*’ were larger than its effects on meta-*d*’, indexed by a decrease in *M*-diff, showing that catecholaminergic enhancement disproportionally increased perceptual over metacognitive performance in absolute terms (Fig. 1*F*). Note, however, that the relative difference between *d*’ and meta-*d*’ (*M*-ratio) was not significantly affected by ATX (Fig. 1*G*). These effects were conceptually replicated by measurements of prestimulus pupil size, an indirect proxy of baseline neuromodulator levels. Specifically, prestimulus pupil size exhibited a tight relation with *d*’ (quadratic as well as linear; see also Beerendonk et al., 2024), but not with meta-*d*’ (Fig. 1*H*). Similar to the effects of ATX, *d*’ was disproportionally increased in absolute terms as compared with meta-*d*’ during states of high pupil-linked arousal, indicated by a negative linear relation between baseline pupil size and *M*-diff (Fig. 1*H*).

At first sight, these findings may seem to contradict a study by Hauser et al. (2017), who observed that antagonization of noradrenaline via propranolol increased metacognitive performance (quantified as AUROC2), rather than affecting *d*’. However, in their experiment decision accuracy was titrated to ∼71% for each participant on each drug session; therefore, propranolol could not have had any effects on first-order task performance by design. Note that the increase in AUROC2 in the absence of *d*’ effects under propranolol (and the specific experimental setting) is suggestive of increased metacognitive efficiency. Thus, elevated catecholaminergic neuromodulation seems to weaken metacognitive efficiency (Fig. 1*F*, this study) whereas lowered catecholaminergic neuromodulation seems to increase metacognitive efficiency (Hauser et al., 2017). Together, these findings suggest that catecholaminergic neuromodulation controls the optimization of either perceptual or metacognitive performance. This resonates well with theories of cortical functioning (Yu and Dayan, 2003, 2005; Hasselmo, 2006; Hasselmo and Sarter, 2011) and recent empirical findings postulating that increased neuromodulation reduces the relative contribution of top-down processes in favor of bottom-up processes and vice versa (de Gee et al., 2017, 2020; Kang et al., 2014; Krishnamurthy et al., 2017; Lawson et al., 2021).

### Catecholamines affect the accumulation, but not encoding, of sensory information

Analyses of neural data revealed that ATX did not modulate the encoding of visual information (stimulus orientation decoding; Fig. 2), but rather increased the amplitude of the CPP, a marker of evidence accumulation (Fig. 3*B*,*C*). The latter observation is in line with previous work, including our own, showing that catecholaminergic neuromodulation controls sensory evidence accumulation, both at the behavioral level (i.e., drift rate) and neural level (i.e., CPP; Beste et al., 2018; Loughnane et al., 2019; van Kempen et al., 2019; Nuiten et al., 2023). However, the absence of catecholaminergic effects on encoding of sensory information seems to contradict earlier studies reporting enhanced representational precision of sensory input with increased catecholaminergic levels and elevated pupil-linked arousal (Vinck et al., 2015; Warren et al., 2016; Podvalny et al., 2021). One fMRI study observed that ATX increased representational precision of visual categorical (objects, houses, faces) information in the ventral visual stream (Warren et al., 2016). We recorded neural activity with EEG, a recording method that (contrary to fMRI) excels in temporal precision but not spatial specificity, which may have obscured localized effects of representational precision. Also, because we used Gabor patches we targeted mostly processing in early visual cortex (Hubel and Wiesel, 1962), as opposed to ventral object-level representations (Riesenhuber and Poggio, 1999; Iordan et al., 2015). As the primary target of ATX, the noradrenaline transporter (NET), has relatively low concentrations in early sensory cortices and is more highly concentrated in the central and frontal regions, ATX may have differentially affected the processing of low- versus high-level visual features (Hansen et al., 2022). The relatively high concentration of NET in the central and frontal regions could also explain the prominent ATX-related CPP and FP modulations measured over the parietal and frontal cortex, respectively.

### Relating catecholaminergic effects on behavioral and neural markers of metacognition

To assess how elevated neuromodulator levels affected metacognitive decision-making at the neural level, we computed the FP component, which has previously been suggested to be a marker of metacognitive accuracy (i.e., the congruency between decision accuracy and confidence; Lim et al., 2020; Feuerriegel et al., 2022). In the placebo condition, preresponse FP amplitude did not reflect confidence or accuracy separately (the CPP did) but was less negative for metacognitively correct versus incorrect decisions (Fig. 3*E*, right panel). This extends previous work in humans, showing that activity in a network of the medial and lateral prefrontal regions correlates with metacognitive evaluations (Yokoyama et al., 2010; Vaccaro and Fleming, 2018). It has been proposed that prefrontal networks may support metacognition by estimating the uncertainty of neural representations of sensory input (Geurts et al., 2022). According to this idea, an appropriate assessment of representational uncertainty will result in high confidence for low sensory uncertainty, and low confidence for high sensory uncertainty, and thus appropriate metacognitive evaluations. Speculatively, the FP component may reflect whether prefrontal uncertainty estimation was successful, although this suggestion requires further substantiation. Interestingly, the relation between metacognitive accuracy and preresponse FP amplitude that we observed during placebo and for trials with small baseline pupils was inverted under ATX and trials with large baseline pupils. If the prefrontal regions indeed estimate the uncertainty of neural representations of sensory input, and the FP reflects whether this process was successful or not, the inversion of metacognitive accuracy representations in FP amplitude under elevated neuromodulatory levels may indicate that prefrontal networks were less well able to construct metacognitive evaluations about task performance. This would be in line with the observations that *M*-diff is lowered under ATX and on trials with large prestimulus pupils, as these findings suggest a relative reduction in the accuracy of metacognitive decisions. Prefrontal networks are known to reconfigure under low levels of arousal/neuromodulation during perceptual decision-making tasks (Canales-Johnson et al., 2020; Jagannathan et al., 2022), but whether such reconfigurations affect metacognitive processes needs to be addressed in future work.

### Comparison of the behavioral effects of atomoxetine and pupil-linked arousal

The effects of ATX on behavior were mirrored in the relation between prestimulus pupil-linked arousal and behavior. Specifically, both ATX and prestimulus pupil-linked arousal modulated *d*’, but had no effects on meta-*d*’. In contrast, decision confidence was not affected by atomoxetine, but it did scale with prestimulus pupil-linked arousal (both linearly and quadratically). There may be (at least) two reasons for the discrepancy between ATX and pupillary effects on decision confidence. First, the relation between prestimulus pupil size and confidence was less (statistically) robust than its relation with perceptual sensitivity, and this may indicate that, in general, neuromodulator activity affects perceptual sensitivity more strongly than overall decision confidence. Given that the effects of ATX on *d*’ were already relatively subtle, effects of ATX on decision confidence may have simply been too weak (or may as well be nonexistent). Second, pupil size is affected by the activity of several neuromodulators, e.g., catecholamines, acetylcholine, but also serotonin (Reimer et al., 2016; Larsen and Waters, 2018), all of which having widespread neuromodulatory projections throughout the cortex (Joshi et al., 2016). ATX, as mentioned above, primarily targets the NET, which is not homogeneously distributed throughout the cortex but strongly concentrated over central and frontal cortical regions (Hansen et al., 2022). Thus, ATX and pupil dynamics differ with respect to the involved neuromodulator systems and cortical distribution profiles, which together may underlie their differential effects on decision confidence.

### Catecholaminergic effects fit with parallel and hierarchical models of perceptual metacognition

Although this study was not set out to test the validity of various models of metacognition, the results seem most consistent with the so-called dual-channel and hierarchical models, over first-order models of confidence formation. First, typically, both dual-channel and hierarchical models of metacognition propose that perceptual inference and metacognitive inference are sequential processes, each corrupted by specific sources of noise (i.e., sensory or metacognitive noise). For instance, under hierarchical models, the perceptual decision is affected by sensory noise, whereas the metacognition decision is—given the sequential nature of these processes—affected by both sensory and metacognitive noise. Consequently, a relative decrease of sensory noise over metacognitive noise disproportionally enhances perceptual performance over metacognitive performance, resulting in worsened metacognitive efficiency (note that overall confidence levels also increase under this scenario; J. W. Bang et al., 2019). Indeed, empirical work demonstrated that minimizing sensory noise, through perceptual learning, simultaneously improves perceptual performance and decreases metacognitive efficiency (J. W. Bang et al., 2019). Similarly, here we showed that elevated catecholaminergic neuromodulation disproportionally increased perceptual sensitivity over metacognitive sensitivity (indexed by lower *M*-diff, Fig. 1*F*). Note, however, that our results may also be explained as modulations of two independent processes, as proposed by dual-channel models of metacognition.

Second, perceptual and metacognitive decisions are thought to be supported by, respectively, centroparietal (Gold and Shadlen, 2007; O’Connell et al., 2012; Kelly and O’Connell, 2013; Shadlen and Kiani, 2013; Nuiten et al., 2023) and (pre)frontal cortices (Rounis et al., 2010; Fleming et al., 2012b; D. Bang and Fleming, 2018; Di Luzio et al., 2022). For example, causally interfering with activity in the anterior (a)PFC via TMS selectively enhances metacognitive efficiency, possibly due to a decrease in metacognitive noise (Shekhar and Rahnev, 2018). Furthermore, oscillatory activity in the theta-band (4–8 Hz) over aPFC reflects metacognitive but not sensory accuracy (Wokke et al., 2017). Accordingly, we observed differential effects of catecholaminergic neuromodulation over the centroparietal and frontal regions. ATX increased the amplitude of the CPP, an ERP component that increased in amplitude for correct versus incorrect and high versus low confidence decisions, but did not reflect metacognitive accuracy (congruency between accuracy and confidence; Fig. 3*B*). Conversely, FP amplitude was not modulated by decision accuracy or confidence but was more negative preresponse for metacognitively incorrect decisions compared with metacognitively correct decisions during the PLC and DNP sessions (Fig. 3*D*). Crucially, this pattern was inversed under ATX, as demonstrated by the three-way interaction between ATX/PLC, accuracy, and confidence (Fig. 3*D*). These findings provide novel insight into how parallelly or hierarchically structured inferential processes are associated with electrophysiological patterns of brain activity, facilitating further insight into how they might be implemented in cortex.

### Absence of consistent cholinergic effects

DNP suppressed FP amplitude at relatively early points in time but did not produce other consistent effects in terms of behavior and neural activity. Previous work, using the same dosage of DNP (5 mg orally), has observed mixed effects of this pharmaceutical. First, we have previously reported that DNP slightly increased algorithmic nondecision time and appeared to modulate neural activity in opposite directions of ATX, e.g., ATX increased CPP peak amplitude, whereas DNP suppressed CPP peak amplitude (Nuiten et al., 2023). Two other studies have observed that DNP had effects on neural activity, but only during resting-state (nontask related) measurements. For instance, Pfeffer et al. (2018, 2021) observed that DNP lowered occipital alpha band (8–12 Hz) power and reduced cortex-wide inter-regional functional connectivity during resting-state measurements, but not during experiments requiring a behavioral response. Taken together, this may suggest that 5 mg of donepezil has limited effects on perceptual decision-making behavior, although it may affect cortical activity and connectivity at rest. Pfeffer et al. (2018) speculated that this may be related to the fact that behaviorally relevant cholinergic activity largely consists of phasic transients (by, e.g., modulating neural gain), rather than through long-lasting tonic effects (a similar argument has been made by Sarter and Lustig, 2020). If this is indeed the case, it may be difficult to causally assess the relation between the cholinergic system and human behavior, as methods for interfering with the cholinergic system are currently limited to pharmacology. Note, however, that animal work—using, e.g., optogenetic stimulation of the basal forebrain—demonstrated that the cholinergic system is involved in shaping sensory processing (Herrero et al., 2008; Soma et al., 2012, 2013; Pinto et al., 2013).

### Open questions and future directions

We observed that *d*’ was higher during ATX and on trials with enlarged prestimulus pupil size, whereas meta-*d*’ was not. We directly subtracted *d*’ and meta-*d*’ to test the interaction between perceptual and metacognitive sensitivity effects (resulting in *M*-diff, reflecting metacognitive efficiency), which is possible because these variables are expressed in the same scale. Therefore, we could conclude that the improvement in *d*’ is not only significant in itself but also significantly larger than the null effect observed for meta-*d*’ (Nieuwenhuis et al., 2011; Meyen et al., 2022). Metacognitive efficiency (often quantified as *M*-diff: meta-*d*’ – *d*’ or *M*-ratio: meta-*d*’ / *d*’) is thought to reflect metacognitive performance irrespective of first-order task performance (Fleming and Lau, 2014). This is based on the core assumption that *d*’ and meta-*d*’ generally scale proportionally and that when meta-*d* lags behind *d*’, metacognitive information is “lost.” When that happens (e.g., under ATX), metacognitive evaluations thus actually worsen. This interpretation is in line with our FP results, indicating impoverished frontal EEG signals under ATX, reflecting decreased metacognitive accuracy. Recent work questions to what extent metacognitive efficiency is in fact independent of first-order task performance (Guggenmos, 2021; Rausch et al., 2023) as it may even be influenced by response caution and thus not solely reflect metacognitive capacity (Desender et al., 2022). However, in a hierarchical Bayesian analysis computing *M*-ratio (instead of *M*-diff), we did not observe significant drug effects on *M*-ratio or a relation between prestimulus pupil size and *M*-ratio. Because the *M*-diff and *M*-ratio results were not in line, and consequently no firm conclusions could be drawn with relation to metacognitive efficiency, we have committed to the more conservative interpretation of the results, namely, that neuromodulation improved *d*’, but not meta-*d*’. Whether metacognitive evaluations, corrected for performance improvements (*M*-diff/*M*-ratio), are in fact impoverished by ATX and during high pupil-linked arousal could be further evaluated in the future, for example, by designing experiments in which first-order task performance is equalized between conditions (staircasing within conditions) and then directly evaluate the effect on meta-*d*’, an approach that has been successfully implemented in previous experiments (Allen et al., 2016; Hauser et al., 2017).

## Synthesis

Reviewing Editor: Niko Busch, Westfalische Wilhelms-Universitat Munster Decisions are customarily a result of the Reviewing Editor and the peer reviewers coming together and discussing their recommendations until a consensus is reached. When revisions are invited, a fact-based synthesis statement explaining their decision and outlining what is needed to prepare a revision will be listed below. The following reviewer(s) agreed to reveal their identity: Florent Meyniel. Note: If this manuscript was transferred from JNeurosci and a decision was made to accept the manuscript without peer review, a brief statement to this effect will instead be what is listed below.

# Synthesis

Thank you for submitting the revised version of the manuscript. The reviewers and I have evaluated your manuscript, and I am happy to let you know that we find the manuscript suitable for publication once a few remaining comments have been addressed, and I encourage you to carefully address both of these points.

Specifically, it appears that the finding based on the hierarchical Bayesian analysis, which found "that M-ratio was likely not affected by either drug", did not seem to have affected the study's conclusions. Please clarify.

Finally, I would like to ask you to consider making the data and code publicly available.

# Reviewer 1

My only major concern was in the exposition of the study. The revision has successfully addressed them. In addition, I found the new analyses and changes made in response to the other reviewers have significantly improved the paper.

# Reviewer 2

I have reviewed this manuscript before (R1). In my previous review I praised the authors for an extensive and impressive set of analyses. However, I was concerned about the robustness of the finding that the effect was present for perceptual but not for metacognitive performance. Partly in response to this (and as suggested by another reviewer) the authors have used a hierarchical computation of m-ratio, the results of which did not show a difference. Given that the hierarchical measure is more powerful I tend to trust this measure more, however while the authors added these results to the manuscript it hasn't really affected their conclusions. So, what is now the conclusion of this paper?

A second concern that was raised is that the rationale of pupil analyses was unclear. Given that ATX affects pupil size it seems to me that the rationale was to see whether the effect of ATX on (meta)cognitive performance is mediated by pupil size. Instead, the authors carefully explained that this was _not_ the case and that they took care to avoid this intepretation. While this explanation was clear, still the rationale of this analysis was unclear to me. Perhaps my intuition about the mediation idea is misguided?

## Author Response

Dear Editor of eNeuro, Please find enclosed the second revision of our manuscript "Pharmacological elevation of catecholamine levels improves perceptual decisions, but not metacognitive insight". We have addressed the final comments of Reviewer #2, through minor additions to the manuscript. Below, we provide our detailed response (in black) to the reviewer comments (in blue).

Upon final acceptance, we will publish all data and analyses scripts online at FigShare.

Yours sincerely, and on behalf of all co-authors, Simon van Gaal, Stijn Nuiten Reviewer #2 comments:

In my previous review I praised the authors for an extensive and impressive set of analyses. However, I was concerned about the robustness of the finding that the effect was present for perceptual but not for metacognitive performance. Partly in response to this (and as suggested by another reviewer) the authors have used a hierarchical computation of m-ratio, the results of which did not show a difference. Given that the hierarchical measure is more powerful I tend to trust this measure more, however while the authors added these results to the manuscript it hasn't really affected their conclusions. So, what is now the conclusion of this paper? The newly added M-ratio results (Bayesian hierarchical calculation) have not altered our previous conclusions, because we were initially already conservative in our conclusions. Specifically, we concluded that catecholaminergic neuromodulation improves perceptual decision-making but not metacognitive insight. Below, we will reiterate our results to substantiate this conclusion. We observed that atomoxetine increased d', without affecting meta-d'. Next, we assessed whether the effect of atomoxetine was also different on d' than on meta-d'. The reason for doing this is one the one hand purely statistical (when comparing two effects one must calculate their interaction; Nieuwenhuis et al., 2011) and on the other hand related to the idea that meta-d' may scale with d' and therefore the difference (M-diff) or ratio (M-ratio) between these two sensitivity measures should be a more uncontaminated measure of metacognitive capacities (so-called metacognitive efficiency; Fleming &Lau, 2014). We observed that atomoxetine significantly lowered M-diff, but did not affect M-ratio (although the effect was in the same direction). Thus, we refrained from concluding that atomoxetine in fact impoverished metacognition throughout the entire manuscript. Instead, we conservatively concluded that atomoxetine improved perceptual decision-making without affecting metacognition. This conclusion was therefore based on the observations that 1) atomoxetine increased d', but not meta-d', 2) that the effect of atomoxetine on d' was larger than its effect on meta-d' (interaction effect, M-diff), and 3) that effects of atomoxetine on metacognitive efficiency were inconclusive (no effect M-ratio, positive effect M-diff). Note that the same logic applies to the pupillometry analyses. To further stress that we did not find evidence for an effect of neuromodulator activity on metacognitive efficiency, we have rewritten the following part of the Discussion.

Pages 29-30, lines 974-987 However, in a hierarchical Bayesian analysis computing M-ratio (instead of M-diff), we did not observe significant drug effects on M-ratio nor a relation between pre-stimulus pupil size and M-ratio. Because the M-diff and M-ratio results were not in line, and consequently no firm conclusions could be drawn with relation to metacognitive efficiency, we have committed to the more conservative interpretation of the results, namely that neuromodulation improved d', but not meta-d'. Whether metacognitive evaluations, corrected for performance improvements (M-diff/M-ratio), are in fact impoverished by ATX and during high pupil-linked arousal could be further evaluated in the future, for example by designing experiments in which first-order task performance is equalized between conditions (staircasing within conditions) and then directly evaluate the effect on meta-d', an approach that has been successfully implemented in previous experiments (Allen et al., 2016; Hauser et al., 2017).

A second concern that was raised is that the rationale of pupil analyses was unclear. Given that ATX affects pupil size it seems to me that the rationale was to see whether the effect of ATX on (meta)cognitive performance is mediated by pupil size. Instead, the authors carefully explained that this was _not_ the case and that they took care to avoid this intepretation. While this explanation was clear, still the rationale of this analysis was unclear to me. Perhaps my intuition about the mediation idea is misguided? Our apologies if the intention of the pupillometry analysis was not clear. We indeed did not mean to use baseline pupil size measurements to test if the effects of atomoxetine (and donepezil) were mediated through changes in pupil size. Instead, we wondered whether more spontaneously occurring fluctuations in neuromodulatory drive, reflected in pre-stimulus pupil size, would also show a relation with d', but not with meta-d'. The reasoning for this correlational approach was similar to the causal manipulation of catecholaminergic and cholinergic activity, namely, to see if changes in arousal state (controlled by neuromodulator activity and reflected in pupil size) would differentially relate to perceptual decision-making and metacognition. Through our elaborate binning procedure, we furthermore ensured that spontaneous fluctuations in pupil-linked arousal were orthogonal from the causal pharmacological manipulation of neuromodulator levels. The pupillometry analyses confirmed that spontaneous fluctuations in neuromodulator activity, which occur naturally in humans also in the absence of any pharmacological manipulation, affect perceptual decision-making behavior but not metacognition. We have read through the manuscript carefully and hope to have clarified this approach better and to have avoided any suggestion of a mediation analysis.

References Fleming, S. M., &Lau, H. C. (2014). How to measure metacognition. Frontiers in Human Neuroscience, 8. https://www.frontiersin.org/articles/10.3389/fnhum.2014.00443 Nieuwenhuis, S., Forstmann, B. U., &Wagenmakers, E.-J. (2011). Erroneous analyses of interactions in neuroscience: A problem of significance. Nature Neuroscience, 14(9), 1105-1107. https://doi.org/10.1038/nn.2886
